# Riluzole attenuates acute neural injury and reactive gliosis, hippocampal-dependent cognitive impairments and spontaneous recurrent generalized seizures in a rat model of temporal lobe epilepsy

**DOI:** 10.3389/fphar.2024.1466953

**Published:** 2024-10-30

**Authors:** Thomas Kyllo, Dominic Allocco, Laine Vande Hei, Heike Wulff, Jeffrey D. Erickson

**Affiliations:** ^1^ Neuroscience Center of Excellence, School of Medicine, Louisiana State University Health-New Orleans, New Orleans, LA, United States; ^2^ Department of Pharmacology, School of Medicine, University of California-Davis, Davis, CA, United States

**Keywords:** acute neural injury, kainic acid, neuroprotection, neuroinflammatory response, microglia and astrocytes, epileptogenesis, antiepileptogenic drug

## Abstract

**Background:**

Riluzole exhibits neuroprotective and therapeutic effects in several neurological disease models associated with excessive synaptic glutamate (Glu) release. We recently showed riluzole prevents acute excitotoxic hippocampal neural injury at 3 days in the kainic acid (KA) model of temporal lobe epilepsy (TLE). Currently, it is unknown if preventing acute neural injury and the neuroinflammatory response is sufficient to suppress epileptogenesis.

**Methods:**

The KA rat model of TLE was used to determine if riluzole attenuates acute hippocampal neural injury and reactive gliosis. KA was administered to adult male Sprague-Dawley (250 g) rats at 5 mg/kg/hr until status epilepticus (SE) was observed, and riluzole was administered at 10 mg/kg 1 h and 4 h after SE and once *per* day for the next 2 days. Immunostaining was used to assess neural injury (FJC and NeuN), microglial activation (Iba1 and ED-1/CD68) and astrogliosis (GFAP and vimentin) at day 7 and day 14 after KA-induced SE. Learning and memory tests (Y-maze, Novel object recognition test, Barnes maze), behavioral hyperexcitability tests, and spontaneous generalized recurrent seizure (SRS) activity (24-hour video monitoring) were assessed at 11–15 weeks.

**Results:**

Here we show that KA-induced hippocampal neural injury precedes the neuroimmune response and that riluzole attenuates acute neural injury, microglial activation, and astrogliosis at 7 and 14 days. We find that reducing acute hippocampal injury and the associated neuroimmune response following KA-induced SE by riluzole attenuates hippocampal-dependent cognitive impairment, behavioral hyperexcitability, and tonic/clonic generalized SRS activity after 3 months. We also show that riluzole attenuates SE-associated body weight loss during the first week after KA-induced SE.

**Discussion:**

Riluzole acts on multiple targets that are involved to prevent excessive synaptic Glu transmission and excitotoxic neuronal injury. Attenuating KA-induced neural injury and subsequent microglia/astrocyte activation in the hippocampus and extralimbic regions with riluzole reduces TLE-associated cognitive deficits and generalized SRS and suggests that riluzole could be a potential antiepileptogenic drug.

## Introduction

Temporal lobe epilepsy (TLE) is the most common epileptic syndrome in adults and is characterized by focal seizures originating from or involving one or both of the temporal lobes (Prince, 1985; Prince, 1993; Engel, 2001). TLE can develop secondary to an initial brain insult such as trauma, infection, or status epilepticus (SE) (Vezzani et al., 2016; Pujar et al., 2018; Fordington and Manford, 2020; Bodendiek et al., 2023). The initial insult is followed by a latent period (i.e., epileptogenesis), after which spontaneous recurrent seizure (SRS) activity occurs, which many believe to be due to a hyperexcitable neural network that has developed in limbic circuits of the brain that leads to hippocampal sclerosis (Pitkanen and Lukasiuk, 2011; Lukawski et al., 2018; Marques et al., 2022). Cognitive impairments, specifically involving learning and memory, along with increased depression and other mood disorders are also common features of adults with epilepsy (Heilman, 2021; Novak et al., 2022). While there has been significant progress in research and treatment over the past few decades, epilepsy still affects 1% of people globally and > 30% of patients with epilepsy respond poorly to pharmacological therapy (French, 2007; Fattorusso et al., 2021). Existing anti-seizure medications cannot address all the pathological features and comorbidities such as hippocampal sclerosis (neuronal loss), neuroinflammation, synaptic reorganization, and cognitive/behavioral impairments that occur in patients with epilepsy (Dudek and Staley, 2011; Kaminski et al., 2014). Medications that prevent epileptogenesis (i.e., the development of epilepsy) and modify epileptic disease progression in patients with increased risk after an initial SE event are not well represented in the clinic (Marson et al., 2005; Lukawski et al., 2018; Galanopoulou et al., 2021).

Rodent models of TLE are valuable resources to evaluate potential antiepileptogenic drugs that prevent hippocampal neural injury and neuroinflammation because epilepsy develops in rodents in weeks to months, while in humans decades after initial SE events are sometimes required (Becker, 2017). Rodent models can be useful to understand mechanisms involved in TLE pathology including acute neural injury that may be targeted to prevent epileptogenesis (Loscher, 2020; Benassi et al., 2021). Systemic administration of the glutamate (Glu) analog kainic acid (KA) produces limbic seizures culminating in SE which leads to neuronal loss within the hippocampus and associated limbic structures (Nadler, 1981) in a pattern similar to that observed in TLE (Ben Ari, 1985). KA-induced neurotoxicity is dependent on synaptic release of Glu from hippocampal dentate gyrus (DG) mossy fiber axon terminals onto DG hilar (DG/CA4) interneurons (mossy cells and GABAergic interneurons) and CA3 pyramidal neurons resulting in hyperexcitation of these neurons leading to NMDAR-induced excitotoxicity. Glu-induced excitotoxicity proceeds via CA3 neurons and overactive Schaffer collaterals to the CA1 region and then to connecting excitatory hippocampal output projections. KA-induced excitoxicity also occurs in other limbic areas producing neural injury in multiple regions in the brain that include the entorhinal and piriform cortices, the amygdala, and the mediodorsal thalamus (Sperk, 1994; Levesque and Avoli, 2013; Srivastava et al., 2020; Rusina et al., 2021). A critical gap in our understanding of the triggers of epileptogenesis is whether drugs that prevent acute hippocampal neural injury and neuroinflammation following an initial SE event can prevent epileptogenesis. Activated microglia and astrocytes are thought to be critical drivers of epileptogenesis after acute neural injury (Coulter and Steinhauser, 2015; Wyatt-Johnson et al., 2017; Sano et al., 2021; Hayatdayoudi et al., 2022; Purnell et al., 2023; Henning et al., 2023). Microglia are the resident immune cells of the brain that respond to acute neural injury by providing phagocytotic removal of injured neurons. Microglial activation is well-known to occur in rodent models of brain injury (Donat et al., 2017) that can lead to long-term cognitive deficits (Saba et al., 2021). Astrocytes are critical for neuronal survival and the health of hippocampal excitatory synapses in the brain as they provide nutrients to axon terminals including metabolic tricarboxylic acids (Karagiannis et al., 2021) and amino acids such as alanine and glutamine (Gln) that support neural glutamatergic transmission (Andersen and Schousboe, 2023). Astrocytes are also the major route for detoxification of synaptically released Glu *via* high-affinity Glu transporters (EAATS) that clear the synaptic cleft of Glu to prevent NMDAR excitotoxicity of postsynaptic neurons. The loss of Glu transport by Glt-1 (EAAT2) into astrocytes to maintain low extracellular Glu levels in synapses results in the development of epilepsy (Rothstein et al., 1996; Tanaka et al., 1997).

Riluzole is an anti-glutamatergic neuroprotective agent first described in the late 1990s (Stutzmann et al., 1997) and is thought to reduce synaptic Glu release from axonal synapses by multiple mechanisms (Doble, 1996). Riluzole is an FDA approved drug for amyotrophic lateral sclerosis (ALS); although its efficacy in preventing disease progression is limited (Petrov et al., 2017). Riluzole is reported to attenuate cognitive decline in Alzheimer’s disease models with early synaptic Glu hyperactivity in cortical circuits during the disease process (Gulyaeva, 2020). Riluzole has a neuroprotective effect in an experimental model of traumatic brain injury in rats (Wahl et al., 1997), which also relies on excessive Glu synaptic transmission in hippocampal synapses (Dorsett et al., 2017). A neuroprotective effect of riluzole was also observed in Glu-induced acute noise-induced hearing loss (Ruel et al., 2005). Even though riluzole is an anti-convulsant agent (Mizoule et al., 1985; Borowicz et al., 2004; Kim et al., 2007; Citraro et al., 2023), riluzole is currently not utilized in epilepsy therapy likely because of sedative and mental slowing properties (Romettino et al., 1991; Pittenger et al., 2008). Riluzole’s potential as an antiepileptogenic agent for preventing acute hippocampal neural injury and the neuroimmune response that leads to cognitive impairment, behavioral excitability, and SRS activity has not previously been examined in animal models of epilepsy *nor* in humans.

Recently, we demonstrated for the first time that riluzole prevents KA-induced acute hippocampal neural injury that is normally observed at 3 days when given 1 h after the first KA-induced SE event in rats (Kyllo et al., 2023). Here, we also examine microglial and astrocyte activation in the hippocampus at 7 and 14 days following SE to determine whether attenuating acute hippocampal neural injury by riluzole attenuates acute microglial activation and astrogliosis. Additionally, we investigate if attenuating acute hippocampal neural injury and reactive gliosis prevents TLE-associated cognitive impairment, behavioral hyperexcitability, and generalized SRS activity that occurs after 3 months post KA-induced SE. We also report body weight during the first post-SE week to determine if riluzole attenuates SE-induced weight loss. Our data indicate that riluzole could be a potential early therapeutic intervention to prevent epileptogenesis.

## Materials and methods

### Animals

We used male Sprague-Dawley albino rats (Envigo) for all studies. Adult (9–11–weeks old, ∼225 g) rats were allowed to acclimatize to the Neuroscience Center of Excellence animal care center for 4–7 days and were used for all experiments. All animal procedures were approved by the Institutional Animal Care and Use Committee (IACUC) at LSUHSC, which abides by NIH guidelines for animal research. All measures were taken to minimize pain, discomfort, or suffering of animals. Rats were housed 2 *per* cage in an environmentally controlled room (23°C, 12-h light/12-h dark cycle) with food and water available *ad libitum*. Rats were monitored daily by the Division of Animal Care staff.

### KA-induced SE

**FIGURE 1:**
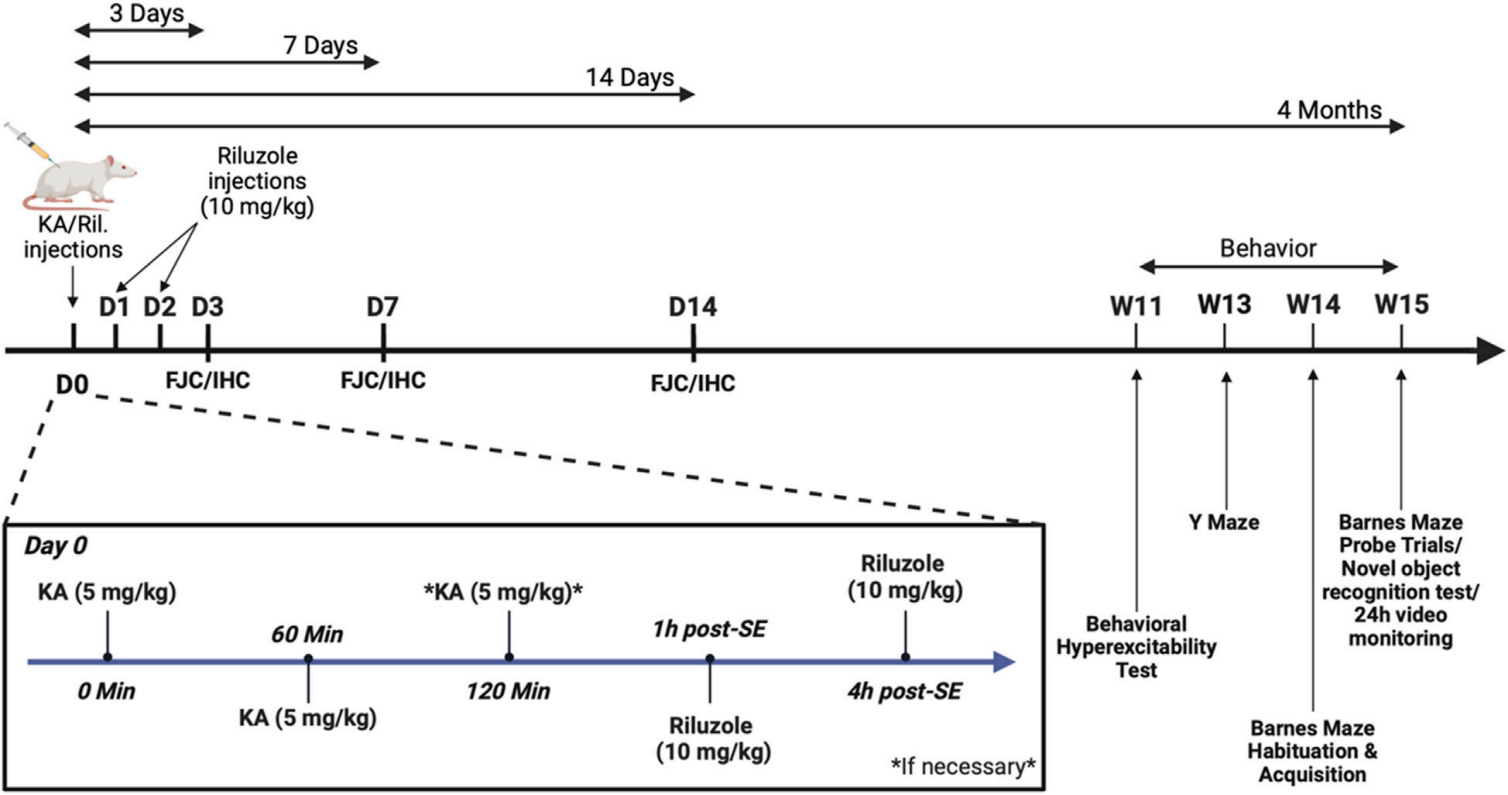
Timeline of the experimental design used for sham, KA-injection and riluzole treatment and the immunohistochemical and behavioral experiments.

Rats (∼250 g) were injected intraperitonially (i.p.) with KA (5 mg/kg/hr) in buffered saline to produce generalized SE (stage 4/5) as previously described (Kyllo et al., 2023). The timeline of the experimental design used for sham, KA-injections and riluzole treatment along with the immunohistochemical and behavioral analyses are shown in Figure 1. Most rats exhibit SE with two KA injections but some required three KA injections (Kyllo et al., 2023). Rats that did not reach SE (<15%) were excluded from the study as before (Kyllo et al., 2023). Mortality is virtually eliminated by KA injections of 5 mg/kg/hr (Hellier et al., 1998). Seizure severity was assessed using the well-established five stage Racine Scale (Racine, 1972): (0) No behavioral change, (1) Mouth and facial movements (orofacial movements), (2) Head nodding (head myoclonus and/or severe orofacial movements), (3) Forelimb clonus, (4) Generalized seizures with rearing and, (5) Generalized seizures with rearing and falling with loss of postural control. Separate groups of rats were treated by i.p. injection of vehicle or riluzole (10 mg/kg; 1 mL; Tocris) 1 and 4 h after the first generalized seizure (SE) was first observed, and then once daily for 2 days as described (Kyllo et al., 2023). Once KA-induced SE was first observed, stage 5 seizures activity occurred repeatedly, and lasted 4–6 h. The seizure activity of rats was monitored for 6 h following initiation of SE. The body weights of sham (saline) rats, KA + vehicle-treated rats, and KA + riluzole-treated rats were observed for 14 days with the first 6 days reported here.

### Morphologic analysis

Morphologic analysis was performed at 3, 7 and 14 days in sham and KA + vehicle-treated rats and 7 and 14 days in KA + riluzole-treated rats. Morphological analysis of KA + riluzole-treated rats at 3 days post-SE was previously reported (Kyllo et al., 2023). Rats were perfusion fixed with 4% paraformaldehyde (PFA), the brains removed and placed in 4% PAF overnight at 4°C, cryopreserved with 15%, then 30% sucrose and frozen at −80°C until processed. Frozen brains were sectioned using a cryostat at 25 μM containing the medial hippocampal region (Bregma −3.3 to −3.8).

### Fluoro-Jade C labeling

We used Fluro-Jade C (FJC; EMD Millipore) to determine neuronal cell injury in the CA fields and DG/hilar region of the hippocampus. Brain sections were mounted onto gelatin coated Superfrost Plus slides (ThermoFisher), dried for 30 min at 37°C and then incubated in 4% PFA vapor for 30 min at 37°C. Slide mounted sections were immersed in a solution of 1% sodium hydroxide in 80% ethanol for 5 min, transferred to 70% ethanol for 2 min followed by deionized water for 1 min. Slides were then added to a solution of 0.06% potassium permanganate for 10 min on a rocker, rinsed in deionized water for 1 min and then transferred to the FJC Working Solution (0.0004% solution dissolved in 0.1% acetic acid) containing 10 μg/mL of 4′,6-diamidino-2-phenylindole (DAPI; Invitrogen) for 20 min. Following staining, slides were rinsed in deionized water (3 × 1 min), dried at 37°C (∼30 min) and cover-slipped with mounting medium DPX (SigmaAldrich).

### Immunohistochemistry (IHC)

Brain sections were fixed with ice cold methanol for 10 min on ice, rehydrated in 1 x PBS, transferred to ice-cold sodium borohydride (0.1% in 1 × PBS) solution for 10 min followed by three rinses with PBS. Sections were then transferred to 12-well plates and incubated for 1 h in 1 mL blocking buffer (3% bovine serum albumin, 10% donkey serum, 0.25% Triton X) in 1 × PBS at room temperature. The buffer was replaced with 1 mL fresh blocking buffer containing primary antibody for NeuN (1:1,000; MAB377; Millipore), Iba1 (1:2,000; 019–19,741; FUJIFILM), ED-1/CD68 (1:300; MAB1435; Millipore), GFAP (1:1,000; PA3-16727; Invitrogen), or vimentin (1:2000; M0725; Dako) and incubated overnight at 4°C. Sections were washed with 1 x PBS-Tween (0.5%) (3 × 1 mL), 1 × TBS-Tween (0.5%) (3 × 1 mL), and 1 x PBS (3 × 1 mL) at room temperature. Sections were then incubated for 30 min in blocking buffer (diluted 3 × fold in 1 × PBS) at room temperature and then incubated in fresh buffer with species-specific and highly cross-adsorbed secondary antibodies coupled to Alexa 488 or Alexa 594 (Molecular Probes), and DAPI (1 μg/mL) for 1 h at room temperature. The sections were washed again as previously described, then mounted onto Fisher Superfrost Plus slides and cover slipped using Prolong antifade mounting medium (Invitrogen).

### Imaging/quantification

Sections were imaged using a Leica (Nussloch) DMRXA automated upright epifluorescence microscope, a Sensicam QE charge-coupled device digital camera (Cooke Corporation), and a filter set suitable for Alexa fluorophores 488 and 594. Images of sham, KA-treated and the riluzole-treated groups were obtained at the same exposure time for FJC (300 ms; *green*), NeuN (400 ms; *red*), Iba1 (400 ms; *red*), ED-1 (400 ms; *green*), GFAP (400 ms; *red*), vimentin (400 ms; *red*) or DAPI (100 ms; *blue*). Quantification of neuronal cell injury by FJC staining, neural survival by NeuN, microglial activity by Iba1/ED-1, and astrogliosis by GFAP/vimentin fluorescence intensity was performed using ImageJ: FIJI. The images were uploaded to FIJI and then were split by color channels into separate images (*blue*, *red or green*). The drawing tool selected fixed areas in the CA3 and CA1 (50,000 pixels). CA4 hippocampal areas and the DG itself were hand drawn from the drawing tool provided and then the fluorescent intensity of that area was quantitated and compared. The background fluorescent intensity was measured by selecting a similar fixed area free of labeled cells. DAPI is only used to visualize and locate hippocampal subregions of the midline hippocampus, especially where fluorescent labeling of specific markers is minimal. The results were then transferred to Microsoft Excel and the Corrected Total Cell Fluorescence (CTCF) was calculated using the formula CTCF = Integrated Density (IntDen) − (Area of Selected Cell × Mean Fluorescence of background reading) for each hippocampal area.

### High-power imaging

Regions of interest (ROI) from the CA1 region of the hippocampus were imaged using an FV1000 confocal system (Olympus) equipped with a 60 × plan fluor objective (NA = 1.42), 405 and 592 nm excitation diodes, and a multi-Argon laser for collection *via* corresponding photomultipliers. Multiple planes were captured *per* ROI to generate z-stacks at a linear and sequential scanning speed of 40 ms/pixel, resolution of 1,024 × 768, and interplanar space of 1.38 microns. Stacks were subsequently merged into maximum-intensity projections, and individual channels were merged using Fluoview (Olympus) and Photoshop 25.0.1 (Adobe).

### Behavioral analysis

Behavioral analysis was performed at 11–15 weeks in sham, KA + vehicle-treated rats and KA + riluzole-treated rats that were blinded to the investigators performing the tests as to the identity of the groups to which the rats belong. Three groups of rats were used with 12–16 animals *per* group as determined based on previous literature studies (O’Leary and Brown, 2013; Grayson et al., 2015; Gawel et al., 2018; Van Den Herrewegen et al., 2019).

### Behavioral hyperexcitability test

A behavioral hyperexcitability test was used to study behavioral changes following KA-induced SE (Rice et al., 1998; Nizinska et al., 2021). The behavioral hyperexcitability test was performed at 11 weeks post KA-induced SE. Four tests were used to determine hyperexcitability differences: *i)* Approach-response test: A pen held vertically is moved slowly toward the face of the animal. Responses were recorded as 1, no reaction; 2, the rat sniffs at the object; 3, the rat moves away from the object; 4, the rat freezes; 5, the rat jumps away from the object; and 6, the rat jumps and attacks the object. *ii)* Touch-response test: The animal is gently prodded in the rump with the blunt end of a pen. Responses were recorded as 1, no reaction; 2, the rat turns toward the object; 3, the rat moves away from the object; 4, the rat freezes; 5, the rat turns toward the touch; 6, the rat turns away from the touch; and 7, the rat jumps with or without vocalizations. *iii)* Finger-snap test: A finger snap several inches above the head of the animal is performed. Responses were recorded as 1, no reaction; 2, the rat jumps slightly (normal reaction); and 3, the rat jumps dramatically. *iv)* Pick-up test: The animal is picked up by grasping him around the body. Responses were recorded as 1, very easy; 2, easy with vocalizations; 3, some difficulty, the rat rears and faces the hand; 4, the rat freezes; 5, difficult, the rat avoids the hand by moving away; and 6, very difficult, the rat behaves defensively, and may attack the hand.

### Y-maze

The Y-maze was used to determine spatial memory deficits in rats associated with damage to the hippocampus (Lalonde, 2002). The Y-maze was performed at 13 weeks post KA-induced SE. The Y maze has three arms enclosed by walls on all sides and no ceiling. Surrounding the maze were visual cues to assist the rat in spatial awareness and there was a camera directly above. Initially, each rat was placed in the “start” arm of the maze. The rats were then allowed to explore the maze for 5 min, with entry into each arm being recorded. Sham rats show increased spontaneous alternation percentage compared to rats with KA-induced hippocampal damage. The number of arm entries and number of alternations were recorded. Each rat was tested on three consecutive days and results were averaged.

### Novel object recognition test (NOR)

The NOR test is based on the understanding that rodents have a natural curiosity to explore new objects (Grayson et al., 2015). The NOR test was performed at 15 weeks post KA-induced SE. The rat is placed in the center of an open field and allowed to habituate for about 5 min. Following the habituation phase is the acquisition phase, where two distinct novel objects are placed in opposite corners of the open field and the rat can freely explore them for 10 min undisturbed. The final phase (retrieval) consists of replacing one of the objects with a new unfamiliar object and allowing the rat to freely explore for 10 min. The interval between the acquisition phase and the retrieval phase is up to 24 h. Unimpaired rats will spend an equal amount of time exploring both objects during the acquisition phase but spend more time exploring the unfamiliar object when introduced during the retrieval phase. For rats with deficits in memory, an equal amount of time will be spent exploring both objects for both the acquisition and retrieval phases.

### Barnes maze

Hippocampal-dependent spatial learning and memory was examined using the Barnes maze as described (O’Leary and Brown, 2013; Gawel et al., 2018; Van Den Herrewegen et al., 2019). The Barnes maze habituation and acquisition phase were performed at 13 weeks and the probe trials were conducted at 15 weeks post KA-induced SE. Rats prefer dark enclosed spaces and tend to avoid bright open areas. The Barnes maze consists of a large circular surface with circular holes surrounding the perimeter with one of the holes containing a dark box allowing the rat to escape the light environment. White noise at 90 decibels was playing overhead. Spatial cues/images are placed around the room to assist the rat in learning which hole offers escape from the stressful environment. The Barnes maze consists of 3 phases: a habituation phase, acquisition phase and finally the probe trials phase. For the habituation phase on day 0, rats were placed in the center of the surface, allowed to freely explore undisturbed for 5 min, and then gently guided to the escape hole. During the acquisition phase (days 1–4), each rat was placed on the surface and allowed to freely search for the escape hole. If the rat had not entered the escape hole within 3 min, it would be gently guided to the hole. Once completely inside, rats were left undisturbed for 2 min. Each rat was tested twice at least 1 h apart for each acquisition day. Probe trials occurred on days 7 and 10 and consisted of one 90 s test with the escape box removed. Spatial learning and memory were assessed by calculating time until animals reach the escape box, with a decrease in time correlating sufficient spatial learning and memory. Search strategy during acquisition phase trials were defined as random, directional, or serial. Directional is defined as a search in which the primary hole distance and primary errors are both less than or equal to four. Serial is defined as a search strategy in which the rat checked holes sequentially around the perimeter of the table with primary errors and hole distance exceeding four. Finally, random is any other search strategy that does not fall within the above two categories.

### Spontaneous generalized seizure activity

We measured spontaneous generalized seizures (stage 4/5) activity of rats by 24-hour video monitoring using a camera system at 15 weeks post KA-induced SE. Rats were placed in separate cages in front of the video camera, recordings were started using Anymaze software, and rats were left unbothered for 24 h. SRS activity was defined as a generalized tonic-clonic seizure or stage 4/5 seizures on the Racine scale. Video recordings were evaluated in sham, KA + vehicle, and KA + riluzole treated rats to record the number of generalized seizures during this period by two individuals that were blinded to the identity of the groups and then averaged together.

### Statistical analysis

Statistical analysis was performed using GraphPad Prism software version 7.0 (GraphPad). Fluorescent intensity of FJC staining and all antibody immunolabeling in the CA1, CA3 and Hilar/CA4 regions are presented visually as representative sections. Mean fluorescent intensity in the CA pyramidal neuron subfields (CA1-4) and in the DG was quantitated. One-way analysis of variance (ANOVA) was performed on these IHC data and Dunnett’s procedures were used to correct for multiple comparisons. Two-way ANOVA was performed on IHC data followed by Tukey’s procedure to correct for multiple comparisons in the timelines of FJC/NeuN staining and microglial and astrocyte activation. Two-tailed t-test was used to compare sham *versus* KA + riluzole-treated rats. Two-way mixed ANOVA followed by Šídák’s procedure was used for the NOR test to correct for multiple comparisons and analyze the results. Barnes maze data were analyzed by two-way mixed ANOVA followed by Dunnett’s procedure to correct for multiple comparisons. Barnes maze search strategy was analyzed using simple linear regression test. Y-maze, behavioral hyperexcitability and generalized SRS activity (stage 4/5) was assessed using one-way ANOVA with Dunnett’s multiple comparison test. A value of *p* < 0.05 was regarded as statistically significantly different.

## Results

### Riluzole attenuates acute neural injury in the hippocampus when given 1 h after KA-induced SE

**FIGURE 2:**
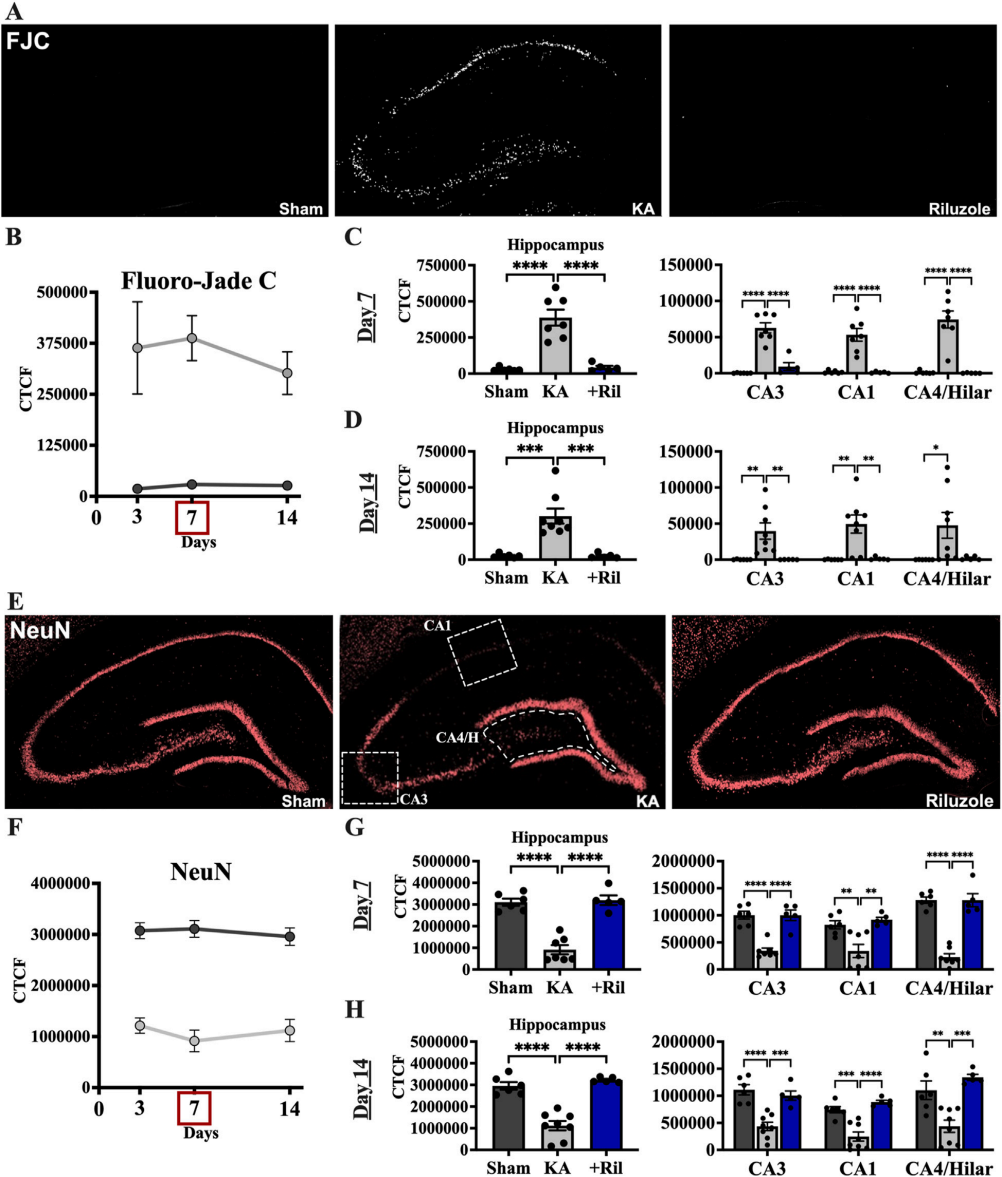
Riluzole attenuates acute hippocampal neural injury during the first 2 weeks when administered after KA-induced SE. **(A)** Representative examples of FJC labeling at 7 days in the entire hippocampus are shown as black and white images. **(B)** Time course of FJC labeling in sham (*dark gray*) and KA + vehicle-treated rats (*light gray*) at 3, 7, and 14 days. **(C, D)** Quantification of the FJC staining in the entire hippocampus and in the CA3, CA1 and CA4/Hilar subfields at 7 days and 14 days, respectively. **(E)** Representative examples of NeuN labeling at 7 days are shown. White boxes represent the areas captured in the quantitative analyses presented. **(F)** Time course of NeuN labeling in sham (*dark gray*) and KA + vehicle-treated rats (*light gray*) at 3, 7, and 14 days. **(G, H)** Quantification of the NeuN immunofluorescence in the entire hippocampus and in the CA3, CA1 and CA4/Hilar subfields at 7 days and 14 days, respectively. All data represent the mean ± SEM (n = 5–8). A value of *p* < 0.05 is regarded as statistically significantly different than values from KA + vehicle-treated rats, with the asterisks corresponding to different levels of significance (*p < 0.05, **p < 0.01, ***p < 0.001, ****p < 0.0001).

Recently we reported that riluzole (10 mg/kg; i.p.) given 1 h after KA-induced SE prevents acute neural hippocampal injury in rats when assessed at 3 days (Kyllo et al., 2023). Here, we tested if neuroprotection by riluzole is maintained 7 and 14 days post-SE using the same protocol for KA-induced SE and drug treatment. We imaged the fluorescence intensity of FJC that labels injured neurons and NeuN that identifies healthy neurons in the entire medial hippocampus and in several hippocampal subfields including the CA3, CA1 and CA4/hilar regions at 7 and 14 days. We observed significant differences in the mean fluorescent intensity between sham and KA + vehicle groups for FJC (F (1,31) = 43.84; *p* < 0.0001) and NeuN (F (1,31) = 146.6; *p* < 0.0001), however there were no differences for either group between timepoints (*FJC*: F (1.379,21.37) = 0.3076; *p* = 0.6571; *NeuN*: F (1.567,24.28) = 0.2258; *p* = 0.7456) (Figures 2A, B, E, F).

Riluzole attenuated neural injury in KA-treated rats at 3, 7 and 14 days in the hippocampus and in all hippocampal subfields. In KA + riluzole-treated rats, FJC staining in the entire hippocampus and in each of the hippocampal CA1-4 neurons was minimal at both day 7 (Dunnett’s: *Hippocampus: p* < 0.0001; *CA3: p < 0.0001; CA1: p* < 0.0001; *CA4/H: p* < 0.0001) and day 14 (Dunnett’s: *Hippocampus: p* = 0.0003; *CA3: p* = 0.0096; *CA1: p* = 0.0049; *CA4/H: p* = 0.0548) and was similar to that observed in sham rats (Figures 2C, D). Riluzole treatment after KA-induced SE also attenuated the diminished NeuN fluorescence normally associated with neural injury after KA-induced SE in all pyramidal neuron subfields of the hippocampus at day 7 (Dunnett’s: *Hippocampus: p* < 0.0001; *CA3: p* < 0.0001; *CA1: p* = 0.0016; CA4/H: *p* < 0.0001) and at day 14 (Dunnett’s: *Hippocampus: p* < 0.0001; *CA3: p* = 0.0006; CA1: *p* < 0.0001; *CA4/H: p* = 0.0003) compared to KA + vehicle rats (Figures 2G, H).

When we compared FJC labeling in sham and KA + riluzole-treated groups we found no significant difference between the two at day 7 (t (9) = 1.087; *p* = 0.3052) and day 14 (t (9) = 0.06365; *p* = 0.9506) (Figures 2C, D). Additionally, we found no difference in the fluorescent intensity of NeuN between sham and KA + riluzole-treated rats at day 7 (t (9) = 0.3328; *p* = 0.7469) and day 14 (t (9) = 1.380; *p* = 0.2009) (Figures 2G, H). These data indicate that riluzole prevents acute neural injury in the CA3, CA1 and CA4/hilar regions of the hippocampus when administered after KA-induced SE.

### Riluzole attenuates acute neural injury in extralimbic regions of the hippocampus

We examined if riluzole also prevents neural injury to other brain regions that are known to be sensitive to excitotoxic Glu transmission after KA-induced SE. We imaged several limbic regions that were present in the same representative slides shown in Figure 2 at days 7 and 14. We find that KA-induced SE results in neural injury in the entorhinal and piriform cortex, the amygdala, and the mediodorsal thalamus as previously documented (Hopkins et al., 2000; Srivastava et al., 2020). Riluzole-treatment showed noticeably less FJC labeling in the entorhinal and piriform cortex, the amygdala, and the mediodorsal thalamus at 7 and 14 days post SE-induced injury indicating neuroprotection in these regions as well (Supplemental data).

### Riluzole attenuates microglial activation at 7 and 14 days post KA induced-SE

**FIGURE 3:**
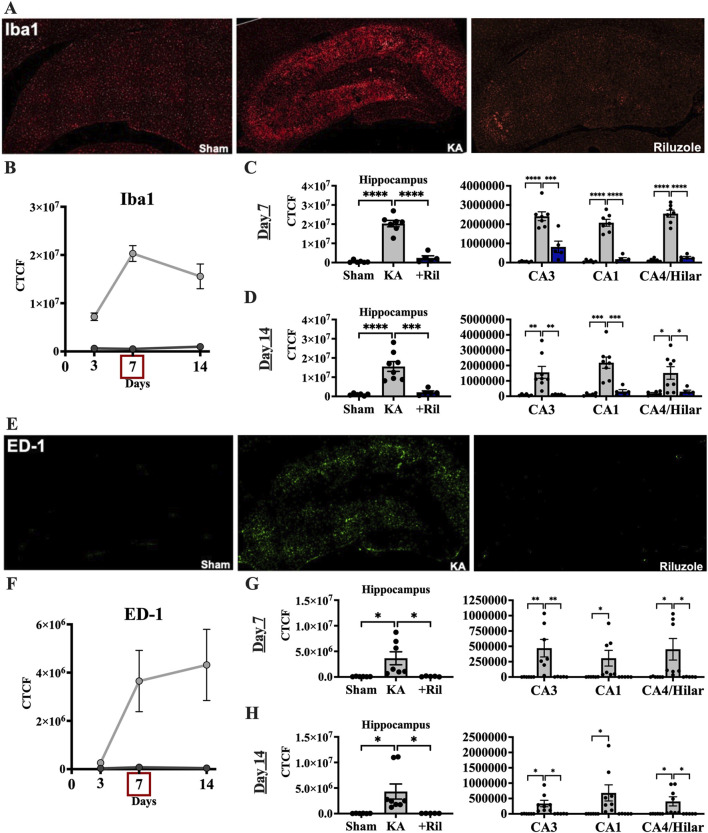
Riluzole attenuates microglial activation after acute neural injury when administered after KA-induced SE. **(A)** Representative examples of Iba1 immunolabeling at 7 days are shown in the entire hippocampus. **(B)** Time course of Iba1 labeling in sham (*dark gray*) and KA + vehicle-treated rats (*light gray*) at 3, 7, and 14 days. **(C, D)** Quantification of Iba1 immunolabeling in the entire hippocampus and in the CA3, CA1 and CA4/Hilar subfields at 7 days and 14 days, respectively. **(E)** Representative examples of ED-1 labeling at 7 days are shown. **(F)** Time course of ED-1 labeling in sham and KA + vehicle-treated rats at 3, 7, and 14 days. **(G, H)** Quantification of the ED-1 immunofluorescence in the entire hippocampus and in the CA3, CA1 and CA4/Hilar subfields at 7 days and 14 days, respectively. All data represent the mean ± SEM (n = 5–8). A value of *p* < 0.05 is regarded as statistically significantly different than values from KA + vehicle-treated rats (*p < 0.05, **p < 0.01, ***p < 0.001, ****p < 0.0001).

We next examined if riluzole could prevent the microglial activation response in the hippocampus that accompanies KA-induced SE and acute hippocampal neuronal injury. We used Iba1 and ED-1 staining in brain sections *in situ* to evaluate levels of KA-induced microglial expression and activation in the hippocampal pyramidal neuron layer subfields. In sham rats, Iba1 labeling is observed uniformly throughout the hippocampus and ED-1 labeling is not present. In KA + vehicle-treated rats, Iba1 fluorescence intensity is greatly increased throughout the hippocampus and specifically in CA regions including CA3, CA1 and CA4/DG subfields (Figure 3E). The timeline for Iba1 and ED-1 expression in the total hippocampus for sham and KA + vehicle groups at 3, 7, and 14 days post SE is shown in Figures 3B, F. We observed significant differences in the mean florescent intensity between the sham group and the KA + vehicle groups for Iba1 (F (1,31) = 101.5; *p* < 0.0001) and ED-1 (F (1,12) = 5.332; *p* = 0.0395). Additionally, we found that Iba1 labeling is significantly increased at day 7 compared to day 3 (F (1.612,24.99) = 7.263; *p* = 0.0053; Tukey’s: *p* = 0.003) (Figure 3B).

In KA + riluzole-treated rats, Iba1 staining in the hippocampal CA1-4 regions was minimal at both day 7 (Dunnett’s: *Hippocampus: p* < 0.0001; *CA3: p* = 0.0002; *CA1: p* < 0.0001; *CA4/H: p* < 0.0001), and day 14 (Dunnett’s: *Hippocampus: p* = 0.0004; *CA3: p* = 0.0057; *CA1: p* = 0.0005; *CA4/H: p* = 0.0276), and similar to that observed in sham rats (Figures 3C, D). Riluzole treatment after SE also attenuated ED-1 fluorescence normally caused by KA in all pyramidal neuron subfields of the hippocampus at day 7 (Dunnett’s: *Hippocampus: p* = 0.0225; *CA3: p* = 0.0085; *CA1: p* = 0.0513; *CA4/H: p* = 0.0360) and day 14 (Dunnett’s: *Hippocampus: p* = 0.0289; *CA3: p* = 0.0162; *CA1: p* = 0.0506; *CA4/H: p* = 0.0399) compared to K + vehicle-treated rats (Figures 3G, H).

When we compared Iba1 levels between sham and KA + riluzole-treated groups we found no significant difference between the two at day 7 [t(9) = 1.944; *p* = 0.0837] and day 14 [t(9) = 1.703; *p* = 0.1227] (Figures 3C, D). We also found no difference in the fluorescent intensity of ED-1 between sham and KA + riluzole-treated rats at day 7 [t(9) = 0.5517; *p* = 0.5946] and day 14 [t(9) = 0.9616; *p* = 0.3614] (Figures 3G, H). These data indicate that riluzole prevents microglial activation following acute neural injury. Higher power images of Iba-1 and ED-1 immunolabeling reveal altered microglial morphology at day 7 after KA-induced SE. Microglia in KA + riluzole-treated rats were similar to those observed in sham rats (Figure 5).

### Riluzole attenuates astrogliosis for up to 2 weeks post KA induced-SE

**FIGURE 4:**
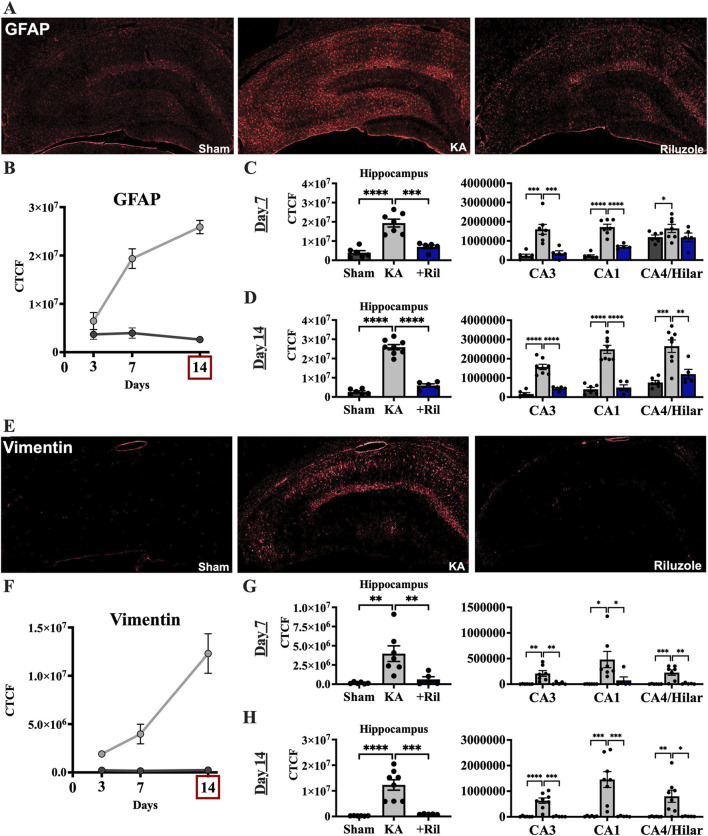
Riluzole attenuates the latent astrocyte response to acute neural injury when administered after KA-induced SE. **(A)** Representative examples of GFAP labeling at 14 days are shown in the entire hippocampus. **(B)** Time course of GFAP labeling in sham (*dark gray*) and KA + vehicle-treated rats (*light gray*) at 3, 7, and 14 days. **(C, D)** Quantification of the GFAP labeling in the entire hippocampus and in the CA3, CA1 and CA4/Hilar subfields at 7 days and 14 days, respectively. **(E)** Representative examples of vimentin labeling at 14 days are shown. **(F)** Time course of vimentin labeling in sham (*dark gray*) and KA + vehicle-treated rats at 3, 7, and 14 days. **(G, H)** Quantification of the vimentin immunofluorescence in the entire hippocampus and in the CA3, CA1 and CA4/Hilar subfields at 7 days and 14 days, respectively. All data represent the mean ± SEM (n = 5–8). A value of *p* < 0.05 is regarded as statistically significantly different than values from KA + vehicle-treated rats (*p < 0.05, **p < 0.01, ***p < 0.001, ****p < 0.0001).

Next, we examined the increase in astrogliosis in the hippocampus that follows acute neural injury and microglial activation. Primary antibodies against astrocytes (GFAP) and activated astrocytes (vimentin) were used. Sections were taken from the same brains as were FJC/NeuN and Iba1/ED-1 (*see* above). In sham rats, minimal GFAP labeling is observed uniformly throughout the hippocampus, while vimentin labeling is not observed (Figures 4A, E). The timelines for GFAP and vimentin expression in the total hippocampus of the sham and KA + vehicle groups at 3, 7, and 14 days post SE are shown in Figures 4B, F. We observed significant differences between the mean florescent intensity of sham and KA + vehicle groups for GFAP [F (1,12) = 105.7; *p* < 0.0001] and vimentin [F (1,12) = 33.13; *p* < 0.0001]. We also observe significantly higher GFAP levels at both day 7 [F (1.827,16.44) = 18.60; *p* < 0.0001; Tukey’s: *p* = 0.0052] and day 14 (Tukey’s: *p* = 0.0032) compared to day 3 (Figure 4B). Vimentin labeling is also highest on day 14 for KA + vehicle treated rats and significantly higher than day 7 (F (1.227,11.66) = 10.68; *p* = 0.0051; Tukey’s: *p* = 0.0344) and day 3 (Tukey’s: *p* = 0.0209; Figures 4B, F).

Riluzole administration after KA-induced SE attenuated GFAP and vimentin expression in KA-treated rats at 14 days in the hippocampus and in all hippocampal subfields. In KA + riluzole-treated rats, GFAP fluorescence intensity was notably decreased throughout the hippocampus particularly in the CA regions including CA3, CA1 and CA4/DG subfields. Vimentin fluorescent labeling in KA + vehicle-treated rats was greatly increased in areas of neural injury (high FJC labeling) and increased Iba1 density above. In KA + riluzole-treated rats we observed uniform GFAP labeling throughout the hippocampus (Figures 4A, E) along with a lack of vimentin labeling (Figure 4E) similar to what we see in sham animals. Quantification of GFAP fluorescent intensity in hippocampal CA1-4 neurons in KA + riluzole-treated rats showed minimal labeling at both day 7 (Dunnett’s: *Hippocampus: p* = 0.0001; *CA3: p* = 0.0008; *CA1: p* < 0.0001; *CA4/H: p* = 0.1670), and day 14 (Dunnett’s: *Hippocampus: p* < 0.0001; CA3: *p* < 0.0001; *CA1: p* < 0.0001; *CA4/H: p* = 0.0035), and was similar to that observed in sham rats (Figures 4C, D). Riluzole treatment after SE attenuated vimentin labeling caused by KA in pyramidal neuron subfields of the hippocampus at both day 7 (Dunnett’s: *Hippocampus: p* = 0.0097; *CA3: p* = 0.0071; *CA1: p* = 0.0442; *CA4/H: p* = 0.0016 and at day 14 (Dunnett’s: *Hippocampus: p* = 0.0002; *CA3: p* = 0.0001; *CA1: p* = 0.0010; *CA4/H: p* = 0.0127) similarly to sham rats (Figures 4G, H).

**FIGURE 5:**
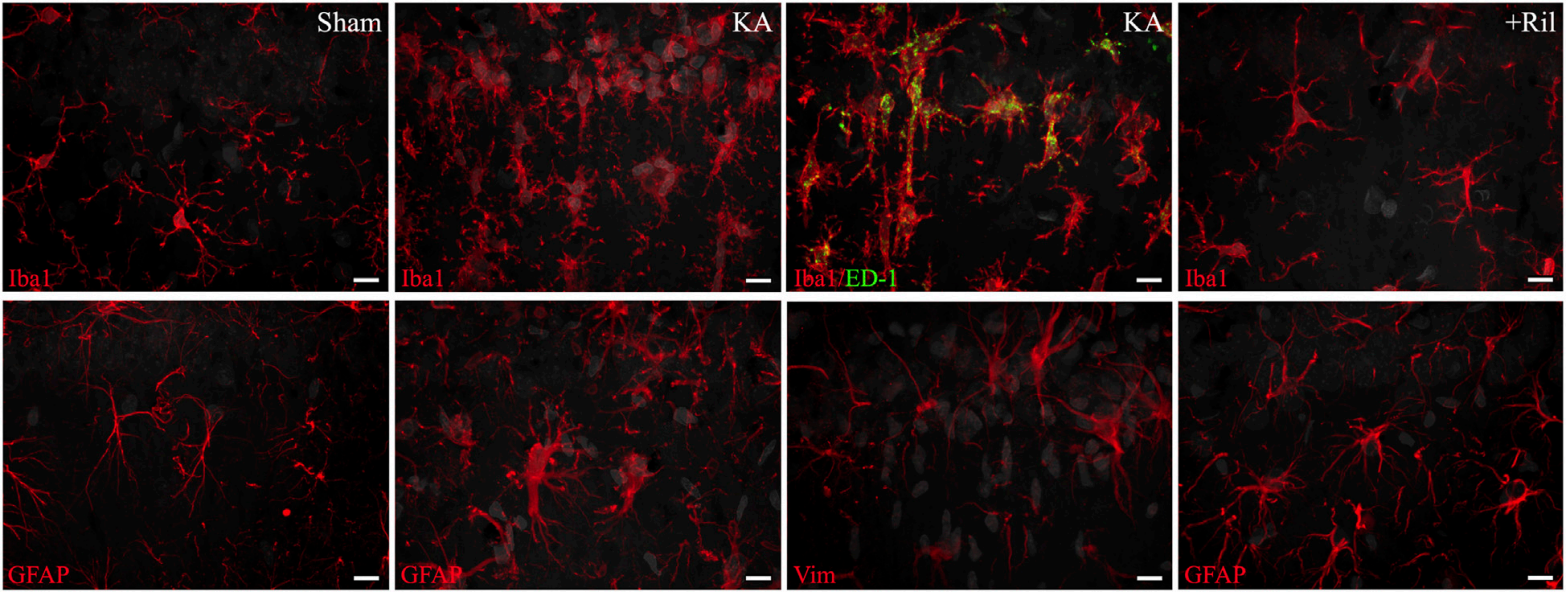
High-power images of Iba1, ED-1, GFAP, and vimentin from the CA1 region of the hippocampus. Iba1 and ED-1 images were taken from slides shown in Figures 2A, E and GFAP and vimentin images were taken from slides shown in Figures 3A, E. The top row shows from *left* to *right*: Iba1 labeling (*red*) in sham, Iba1 labeling (*red*) in KA + vehicle-treated, Iba1 (*red*) and ED-1 (*green*) co-labeling (*yellow*) in KA + vehicle-treated, and Iba1 (*red*) labeling in KA + riluzole-treated rats. The bottom row shows from *left* to *right*: GFAP (*red*) labeling in sham, GFAP (*red*) labeling in KA + vehicle-treated, vimentin (*red*) labeling in KA + vehicle-treated, and GFAP (*red*) labeling in KA + vehicle-treated-treated rats. DAPI stained cell nuclei in the CA1 neurons and in microglia and astrocytes (converted from blue to *gray*) are shown. Scale bar = 10 um. Representative and unbiased images were taken by the Morphology and Imaging Core at LSUHSC-NO.

When we compared GFAP and vimentin labeling between sham and KA + riluzole-treated groups at day 7 and day 14 we found no significant difference between the sham group and KA + riluzole-treated group at day 7 [GFAP: t(9) = 2.011; *p* = 0.0752; vimentin: t (9) = 1.500; *p* = 0.1677]. However, we report that KA + riluzole-treated rats had significantly higher GFAP and vimentin immunofluorescence than sham rats at day 14 [GFAP: t(9) = 3.286; *p* = 0.0094; vimentin: t (9) = 10.39; *p* < 0.0001]; although it was still greatly reduced compared to KA + vehicle rats (Figures 4C, D, G, H). Higher power images of GFAP and vimentin immunolabeling reveal altered astrocyte morphology at day 14 after KA-induced SE. Astrocytes in KA + riluzole-treated rats were similar to those in sham rats (Figure 5).

### Riluzole attenuates KA-induced deficits in hippocampal-dependent learning and memory at 13–15 weeks.

We use the Y maze, NOR test, and Barnes maze to examine hippocampal-dependent recognition, spatial learning and memory after 3 months after KA-induced SE to determine if riluzole can attenuate cognitive impairments associated with epilepsy in this model.

#### Y maze

**FIGURE 6:**
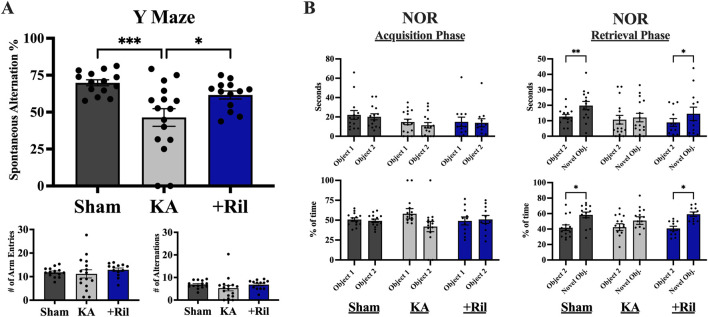
Riluzole improves spatial learning and memory and recognition memory in the KA model of TLE. **(A)** The Y maze was used to test spatial learning and memory in sham, KA + vehicle, and KA + riluzole-treated rats at 13 weeks (n = 12–16). Spontaneous alternation percentage, number of arm entries and number of alternations are shown. Y maze was performed on three consecutive days and data were averaged to get final values. **(B)** KA + riluzole-treated rats exhibit better recognition memory in the NOR test at 15 weeks compared to KA + vehicle-treated rats (n = 12–16). The number of seconds (*top*) and percentage of time spent with each object (bottom) during the acquisition phase (left) and the number of seconds (*top*) and percentage of time (*bottom*) spent with the familiar object and novel object during the retrieval phase (*right*) are shown for sham, KA + vehicle-treated and KA + riluzole-treated rats. Data represent mean seconds and mean percentage of time ±SEM. All scores represent the means ± SEM. A value of *p* < 0.05 is regarded as statistically significantly different than values from KA + vehicle-treated rats (*p < 0.05, **p < 0.01, ***p < 0.001, ****p < 0.0001).

For the Y maze, there was no significant difference between any of the three groups for number of arm entries [F (2,40) = 0.4957; *p* = 0.6128] and number of alternations [F (2,40) = 1.135; *p* = 0.3315] indicating a similar amount of exploratory behavior between sham, KA + vehicle and KA + riluzole-treated groups (Figure 6A). Alternations refer to any three subsequent arm entries (a spontaneous alternation refers to three different arm entries in a row). Spontaneous alternation percentage was determined by dividing the number of spontaneous alternations (rat enters 3 different arms in a row) by the total number of alternations. For spontaneous alternation percentage, KA + riluzole-treated rats explored the novel arm of the maze significantly more than the KA + vehicle-treated group [F(2,40) = 8.151; *p* = 0.0011; Dunnett’s: *p* = 0.0298; Figure 6A], indicative of unimpaired short term spatial memory.

#### NOR test

The NOR test was used to assess the ability of rats to react to spatial changes and is based on the assumption that rats have a natural curiosity to explore novel objects. During the acquisition phase, sham, KA + vehicle, and KA + riluzole-treated groups spent an equal amount of time [F (1,15) = 2.999; *p* = 0.1038] and percentage of time [F (1,15) = 1.662; *p* = 0.2169] interacting with each object (Figure 6B). After one of the objects was replaced with a novel object for the retrieval phase, the KA + vehicle-treated group still spent an equal amount of time (*p* = 0.8466) and percentage of time (*p* = 0.3137) with each object. This is in contrast with both the sham group and KA + riluzole-treated group which spent a significantly more total time (Šídák’s: *sham*: *p* = 0.0035; *riluzole*: *p* = 0.0408) and an increased percentage of time (Šídák’s: *sham*: *p* = 0.0166; *riluzole*: *p* = 0.0181) with the novel object (Figure 6B).

#### Barnes maze test

**FIGURE 7:**
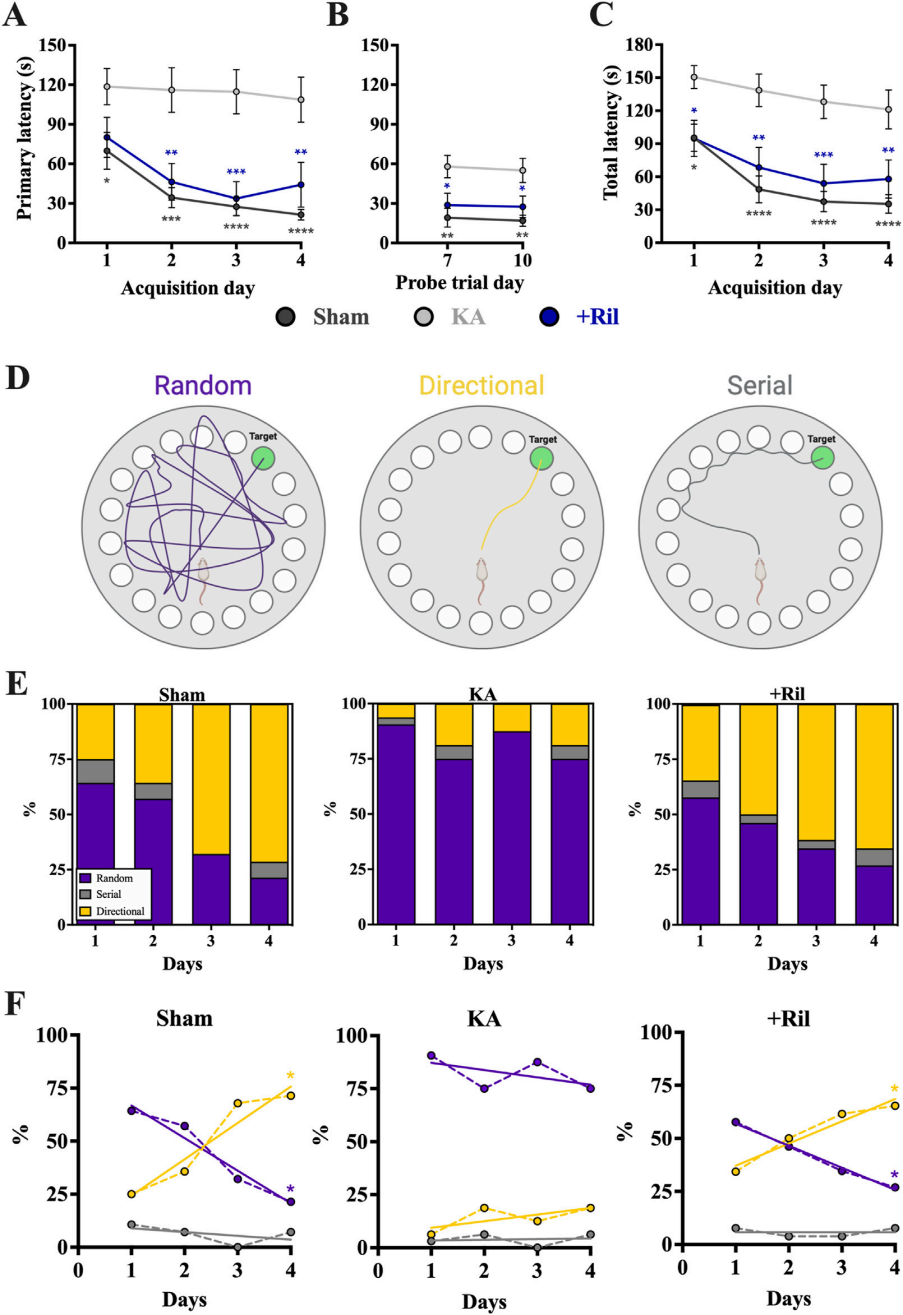
Riluzole attenuates the deficit in spatial learning and memory in the Barnes maze at 15 weeks (n = 12–16). Groups include sham, KA, + vehicle, and KA + riluzole-treated rats. **(A)** Primary latency acquisition days. **(B)** Primary latency probe trial days. **(C)** Total latency acquisition days. **(D)** Schematic representation of the three search strategies: random, directional, or serial. **(E)** Percentage of sham, KA + vehicle-treated and KA + riluzole-treated rats that exhibited each search strategy as random (*purple*), directional (*yellow*), or serial (*gray*) during the acquisition phase. **(F)** Timeline with simple linear regression (*straight lines*) of the shift in search strategy from random to directional for sham and KA + riluzole-treated treated rats compared to KA + vehicle-treated rats that continued to use a random search strategy during the 4 acquisition days. Values represent mean seconds ± SEM. A value of *p* < 0.05 is regarded as statistically significantly different than values from only KA + vehicle-treated rats (*p < 0.05, **p < 0.01, ***p < 0.001, ****p < 0.0001).

The Barnes maze test was used to assess spatial learning and memory deficits, and both primary latency and total latency were measured. Both the sham and KA + riluzole-treated groups showed significant learning and memory during the acquisition phase (days 1–4) as revealed by a decrease in primary latency (Figure 7A). There was a significant main effect of both day [F (3,45) = 7.744; *p* = 0.0003] and treatment [F (2,30) = 13.35; *p* < 0.0001], with the KA + vehicle-treated group performing significantly worse than sham rats on days 1–4 and KA + riluzole-treated rats on days 2–4 (*p* < 0.05). Additionally, the sham and KA + riluzole-treated groups showed significantly lower primary latency than the KA + vehicle group during both probe trial days (Dunnett’s: *p* < 0.05, Figure 7B). During the probe test for spatial memory we find that sham and KA + riluzole-treated rats performed similarly and significantly better than KA + vehicle rats on both day 7 and day 10 trial days. We also observed a significant main effect of both day [F (3,60) = 5.025; *p* = 0.0036] and treatment (F (2,100) = 38.71; *p* < 0.0001) for total latency during the acquisition phase, with the KA + vehicle group performing significantly worse than the sham group and KA + riluzole-treated rats (Dunnett’s: *p* < 0.05, Figure 7C).

#### Barnes maze test search strategy

We also categorized search strategy during the Barnes maze acquisition phase trials as random, directional, or serial (Figure 7D). The fundamental idea behind the Barnes maze test is that unimpaired rats can learn the location of the escape hole. So not only is there a decrease in time it takes rats to find the escape hole each day but a change in the way rats search for the escape hole. Initially a high percentage of rats randomly searched for the escape hole, but as they learned the location of the hole, less random searching was observed and the rats took a more direct route to the hole.

On day 1 a greater percentage of sham rats exhibited a random search strategy (64.29%) than directional search strategy (25.00%). This was significantly different from what we observe on day 4 where the majority of sham rats (71.43%) exhibited a directional search strategy, while only 21.43% exhibit a random search strategy (Figure 7E). This shift in search strategy from predominantly random to predominantly directional [*Random*: F (1,2) = 43.03; *p* = 0.0225; R^2^ = 0.9556; *Directional*: F (1,2) = 21.32; *p* = 0.0438; R^2^ = 0.9142; *Serial* = F (1,2) = 0.7143; *p* = 0.4870; R^2^ = 0.2632] indicated that sham rats exhibited significant spatial learning and memory, specifically with the ability to remember the location of the escape hole (Figure 7F). In the months following KA-induced SE, rats exhibiting SRSs show deficits in spatial learning and memory. In the Barnes maze test this presents as an inability to remember the location of the escape hole. This is consistent with our data that show the KA + vehicle-treated rats do not show a change in search strategy with the overwhelming majority continuing with a random search strategy [*Random*: F (1,2) = 0.8237; *p* = 0.4599; R^2^ = 0.2917; *Directional*: F (1,2) = 1.667; *p* = 0.3258; R^2^ = 0.4545; *Serial*: F (1,2) = 0.03881; *p* = 0.8620; R^2^ = 0.01904; Figures 7E, F]. Similarly to what we see with sham rats, the predominant search strategy for KA + riluzole-treated rats shifted from predominantly random on day 1 (*Random*: 57.69%; *Directional*: 34.32%; *Serial*: 7.69%) to predominantly directional (*Random*: 26.92%; *Directional*: 65.38%; *Serial*: 7.69%) on day 4, indicating that these rats remembered the location of the escape hole [*Random*: F (1,2) = 244.2; *p* = 0.0041; R^2^ = 0.9919; *Directional*: F (1,2) = 30.74; *p* = 0.0310; R^2^ = 0.9389; *Serial* = F (1,2) = 0.000; *p* > 0.9999; R^2^ = 0.000; Figures 7E, F]. This change in search strategy illustrates that KA + riluzole-treated rats took a more direct route to the escape hole on day 4 than on day 1 where they mostly randomly searched for the hole. Together, these data show that systemic administration of riluzole after KA-induced SE leads to improved cognitive deficits, specifically those related to spatial learning and memory.

### Riluzole attenuates behavioral hyperexcitability and spontaneous generalized seizure activity at 11–15 weeks following SE.

#### Behavioral hyperexcitability tests

**FIGURE 8:**
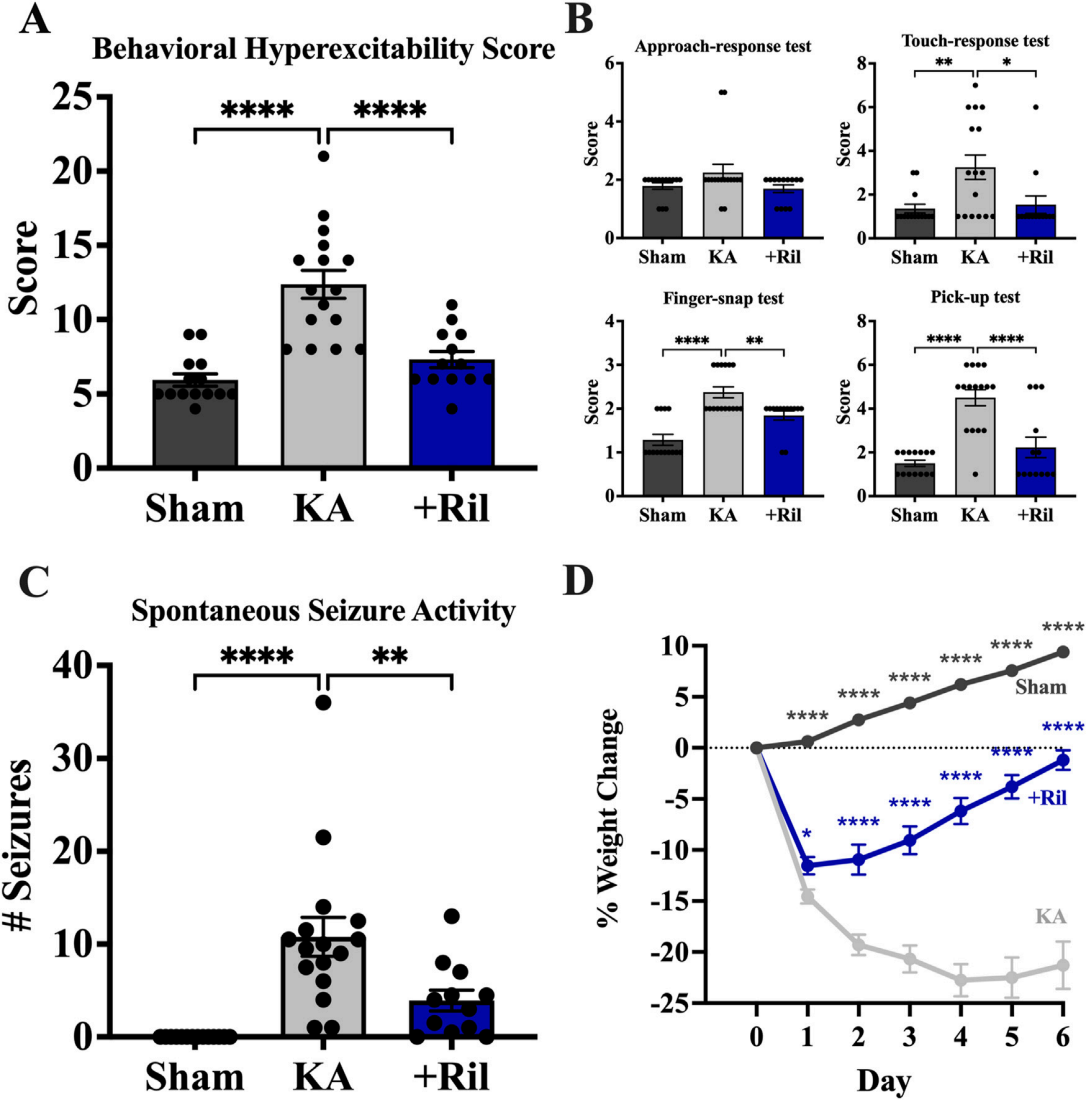
Riluzole treatment reduces behavioral hyperexcitability at 11 weeks. Four separate tests of behavioral hyperexcitability were used; approach-response test, touch-response test, finger-snap test and pick-up test. **(A)** Total behavioral hyperexcitability score was obtained by adding the scores of all 4 tests. **(B)** Scores for approach-response test, touch-response test; finger-snap test, and pick-up test. Scores represent the mean ± SEM. **(C)** Riluzole treatment after KA-induced SE reduces generalized SRS activity at 15 weeks (n = 12–16). Values represent the mean number of generalized stage 4/5 seizures in 24 h period ±SEM. **(D)** KA + riluzole-treated rats show reduced weight loss during the first post-SE week. Percent weight loss of sham rats (n = 24) and KA + vehicle-treated rats (n = 28) and KA + riluzole-treated rats (10 mg/kg; n = 21). A value of *p* < 0.05 is regarded as statistically significantly different than values from KA + vehicle-treated rats (*p < 0.05, **p < 0.01, ***p < 0.001, ****p < 0.0001).

For testing behavioral hyperexcitability in sham, KA + vehicle, and KA + riluzole-treated rats we used 4 behavioral hyperexcitability tests (n = 12–16). Scores of all 4 tests were combined to obtain a Behavioral Hyperexcitability Score, which showed a significant difference between the KA + vehicle group and both the sham group (Dunnett’s: *p* < 0.0001) and KA + riluzole-treated group (Dunnett’s: *p* < 0.0001) (Figure 8A). We observed no difference in the approach response test between any of the 3 groups [F (2, 40) = 2.209; *p* = 0.1231]. Significance differences were observed between KA + vehicle and KA + riluzole-treated groups in the touch-response (Dunnett’s: *p* = 0.0151), finger-snap (Dunnett’s: *p* = 0.0067), and pick-up tests (Dunnett’s: *p* < 0.0001) (Figure 8B).

#### Spontaneous generalized seizure activity

We measured SRS activity as generalized stage 4/5 tonic-clonic seizure events (Racine, 1972) using 24-hour video recordings at 15 weeks. Normally, rats are housed two *per* cage to maintain social interaction but here rats were singly housed during the video recordings. We recorded the total number of spontaneous generalized seizures during a 24-hour period. We used the same rats as we used for the behavioral hyperexcitability tests. The number of generalized seizures recorded for each rat reflect averages from two separate researchers blinded to the groups involved. None of the sham rats exhibited SRS activity. On average, KA + vehicle-treated rats exhibited 10.8 ± 2.1 stage generalized stage 4/5 seizures during the 24-hour period, which is consistent with previous electroencephalogram (EEG) findings (Rusina et al., 2021). In contrast, KA + riluzole-treated rats exhibited significantly fewer seizures with 3.9 ± 1.1 generalized seizures over the 24-hour period (Dunnett’s: *p* = 0.0050; Figure 8C). All KA + vehicle-treated rats exhibited SRS as expected. Two out of 12 KA + riluzole-treated rats did not exhibit SE and three rats only had 1 SE event during the 24-hour monitoring.

#### Riluzole treatment attenuates post-SE body weight loss

Postinjury weight loss during the first post-injury week has been considered as a predictor of epileptogenesis in the lateral fluid percussion induced TBI rat model of posttraumatic epilepsy (Lapinlampi et al., 2020). Weights were monitored for 14 days and days 0–6 are shown here. KA + vehicle-treated rats showed significant weight loss everyday between days 1 and 4 post-SE compared to sham and KA + riluzole-treated rats [F (28,980 = 25.75; *p* < 0.0001)]. Maximum weight loss for KA + vehicle rats was 22.75% and occurred on day 4, while KA + riluzole-treated rats exhibited peak weight loss on day 1 of 11.53%. KA + riluzole-treated rats recovered to their original weight by 1 week. KA + vehicle-treated rats remain suppressed during the first post-SE week. Sham weight levels were continually rising during the course of these experiments (Figure 8D).

## Discussion

It is thought that an effective antiepileptogenic strategy should engage multiple targets that are involved in the development of an epileptic network within the hippocampus because the response to a drug affecting a single target may be inadequate (Lukawski et al., 2018; Loscher, 2020; Marques et al., 2022). Riluzole has multiple identified targets suggesting it may be an ideal antiepileptogenic drug candidate to prevent epilepsy following an acute brain insult. Riluzole could potently both suppress excessive synaptic Glu transmission and prevent Glu excitotoxicity by multiple mechanisms including inhibiting voltage-gated sodium (Na_V_) channels (Herbert et al., 1994; Song et al., 1997; Stefani et al., 1997) to reduce action potential evoked Glu release, the persistent sodium current (I_NaP_) of Na_V_ channels (Urbani and Belluzzi, 2000; Prakriya and Mennerick, 2000; Spadoni et al., 2002) to reduce spontaneous neuronal excitability, voltage-gated calcium (Ca_V_) channels (Huang et al., 1997; Stefani et al., 1997) that are required for Glu exocytosis, neural activity-regulated Gln transport (NARGT) activity (Erickson, 2017; Pietrancosta et al., 2020; Erickson et al., 2023) in axon terminals, and NMDAR’s in postsynaptic neurons (Debono et al., 1993; De Sarro et al., 2000; Citraro et al., 2023). Riluzole could also increase the activity of Ca^2+^-activated potassium channels (Grunnet et al., 2001; Sankaranarayanan et al., 2009) to enhance afterhyperpolarization, and increase clearance of Glu released from synapses on surrounding astrocytes by glutamate transporters such as Glt1/EAAT2 (Fumagalli et al., 2008). Suppression of Glu neural transmission and excitotoxicity *via* these multiple synaptic targets of riluzole could collectively attenuate acute hippocampal neural injury that leads to microglial activation, astrogliosis, and epileptogenesis. Indeed, most of these riluzole targets are known to affect synaptic Glu transmission and these same targets are studied in rodent models of epilepsy (Stasheff et al., 1989; Tani et al., 2007; Blumenfeld et al., 2009; Xu et al., 2013; Kirchheim et al., 2013; Wagnon et al., 2015; Ogiwara et al., 2018; Baker et al., 2018; Khandai et al., 2020; Peterson et al., 2021; Johnson et al., 2022; Lauerer and Lerche, 2023; Berecki et al., 2023).

KA administration results in SE that leads to hippocampal neural injury and a neuroimmune response that initiates epileptogenesis and eventual behavioral and cognitive deficits and the occurrence of SRS. We previously used the KA model of TLE in rats to identify riluzole and several novel aminothiazole derivatives that prevent acute hippocampal neural injury at 3 days when administered after KA-induced SE (Kyllo et al., 2023). Here, we confirm that KA induced hippocampal neural injury occurs by day 3 post-SE and is also present at similar levels at day 7 and day 14, as seen previously (Hopkins et al., 2000). Recent studies also focus on suppressing the neuroimmune response to SE because brain inflammation is thought to be a primary driver of epileptogenesis (Vezzani et al., 2013) and microglial and astrocytes are widely examined as mediators of the neuroimmune response to SE (Shapiro et al., 2008; Coulter and Steinhauser, 2015; Wyatt-Johnson et al., 2017). We found that microglial activation is low at day 3 and maximal by day 7 as assessed by Iba1 and ED-1 immunolabeling. Astrocyte activation was also low at day 3 and became maximal by day 14 post KA-induced SE as assessed by GFAP and vimentin immunolabeling. ED-1/CD68 and vimentin are markers of activated microglial and astrocytes, respectively (Stringer, 1996; Luo et al., 2016). The time course of the progression of microglial and astrocyte activation post-SE in the pilocarpine-induced and diisopropylfluorophosphate-induced chemical models is similar (Shapiro et al., 2008; Flannery et al., 2016) and is consistent with the belief that neuronal injury activates microglia and microglial activation activates astrocytes which then leads to epileptogenesis (Sano et al., 2021; Hayatdayoudi et al., 2022; Henning et al., 2023). High power micrographic data indicate morphologic changes in microglia in the CA1 region at 7 days and in astrocytes at 14 days post KA-induced SE that are consistent with similar changes seen in the pilocarpine model (Shapiro et al., 2008; Wyatt-Johnson et al., 2017), which are not present in KA + riluzole-treated rats in this study. Here, we showed that the short-term use of riluzole (3 days) post KA-induced SE attenuated the microglial and the astrocyte response to acute neural injury up to 14 days.

A critical issue in the field is how to prevent TLE-associated cognitive impairment and SRS activity following acute brain injury or SE (Pitkanen and Lukasiuk, 2011; Benassi et al., 2021; Marques et al., 2022). The development of neuroprotective agents focusing on preventing epileptogenesis is critical to protect the hippocampus against the cascade of events that lead to the reorganization of synaptic circuitry and the development of SRS in an epileptic brain. The results from classic anti-convulsant agents such as phenytoin, phenobarbital, diazepam and carbamazepine to prevent epileptogenesis have been disappointing (Lukawski et al., 2018). While it is possible that neural injury may initiate the epileptogenesis process alone, drugs that promote a neuroprotective effect such as topiramate (a Ca^2+^ channel inhibitor, Ca_V_), for example, did not suppress SRS activity in the pilocarpine-induced SE model (Rigoulot et al., 2004). Levetiracetam, acting through the SV2A vesicular transporter (Sturges et al., 2008), also showed a neuroprotective effect but also did not suppress SRS (Lee et al., 2013). Valproic acid, which acts to influence gene regulation of proteins in Glu and GABAergic pathways may possess antiepileptogenic properties (Lukawski et al., 2018; Romoli et al., 2019) but has negative teratogenic effects (Alsdorf and Wyszynski, 2005). Valproate facilitates GABAergic over glutamatergic transmission (Loscher, 1993; Romoli et al., 2019) and proved to be efficient in inhibiting hippocampal neural injury, behavioral deficits and SRS activity in the KA model (Bolanos et al., 1998). The GABA elevating drug vigabatrin was also neuroprotective in CA1 and CA3 (but not in the DG/CA4 hilar region) after pilocarpine injection and all rats exhibited SRS activity (Andre et al., 2001). The NMDAR antagonist MK-801 attenuates neural injury in the CA1 and CA3 regions but not in the DG/CA4 hilar region of the hippocampus, *nor* in extralimbic regions and especially in the mediodorsal thalamus (Brandt et al., 2003). Likewise, MK-801 did not affect observable generalized SRS when measured after 2 weeks in the KA model (Brandt et al., 2003). The AMPAR antagonist perampanel is a potential anti-epileptic agent (Rogawski, 2013), protects DG/CA4 hilar neurons from performant path stimulation-induced neuronal damage from the entorhinal cortex (Penix and Wasterlain, 1994), and the combination of NMDAR and AMPAR antagonists retards epileptogenesis in the KA model of TLE (Schidlitzki et al., 2017). Increasing astrocyte Glu transporter expression by beta-lactam antibiotics (Rothstein et al., 1995) or ceftriaxone also offers neuroprotection and alleviates cognitive impairments in the pilocarpine model of TLE (Ramandi et al., 2021).

It is possible that a cocktail of drugs that target multiple sites to prevent Glu-induced excitotoxicity and reactive gliosis in the hippocampus and interconnected limbic regions could prevent epileptogenesis following an acute brain insult such as SE (White and Loscher, 2014). On the other hand, we suggest that riluzole could be used alone since it targets most of the sites mentioned above. Additionally, riluzole attenuates acute neural injury in CA1, CA3 *and* in CA4/hilar neurons at 3 days (Kyllo et al., 2023) and up to 2 weeks, attenuates neural injury in extralimbic areas including the mediodorsal thalamus, entorhinal/piriform cortex, and amygdala, and also attenuates acute microglial activation and astrogliosis in the hippocampus following KA-induced SE. We also show here that the short-term use (3 days) of riluzole post KA-induced SE attenuates hippocampal-dependent cognitive impairment, behavioral excitability, and generalized SRS activity measured after 3 months. Protection of CA1 neurons by riluzole could be the simplest explanation of why riluzole produces such a profound improvement of cognition involving spatial recognition, learning and memory. NMDAR antagonism alone cannot prevent KA-induced neural damage in the hippocampal DG/CA4 hilar region *nor* in the mediodorsal thalamus (Brandt et al., 2003), which is also thought to play a critical role in the development of limbic motor seizures (Cassidy and Gale, 1998). The CA4/hilar neurons are also directly targeted by an overactive DG in the KA model and an important issue going forward is to determine which GABAergic neurons in the DG/CA4 hilar region and other CA regions are protected by riluzole (Houser, 2007). As DG/CA4 hilar interneurons in the hippocampus are known to be especially sensitive to KA treatment a selective loss of presumably inhibitory DG/CA4 hilar interneurons may result in hyperexcitability of DG neurons that initiate seizure discharges during the development of epilepsy (Parent and Lowenstein, 1997; Coulter, 2005; Sloviter et al., 2006). It is not clear if riluzole also directly targets sites that may be important to decrease the neuroinflammatory response (Valee et al., 2020; Hodges and Lugo, 2018; Rawat et al., 2023) following KA-induced SE.

Riluzole has previously been shown to reduce seizure activity in multiple *in vivo* seizure models such as a genetic rat model of absence seizure (Romettino et al., 1991), the pilocarpine-induced and the γ-hydroxybutyrate lactone (GBL)-induced absence seizure model (Kim et al., 2007), amygdala kindling (Yoshida et al., 2001), and audiogenic seizures in DBA/2 mice (De Sarro et al., 2000). Recent studies indicate that riluzole suppressed spontaneous firing and increased the action potential firing threshold of pluripotent stem cell-derived neurons from 3 patients with missense variants in the *SCN8A* Na_V_ gene (Tidball et al., 2020). These patients had dramatic reductions in seizure frequency for several months after starting riluzole treatment, but patients elected to discontinue treatment due to the appearance of side effects (excessive sleepiness, urinary tract infections) or loss of efficacy (Wengert and Patel, 2021). Riluzole may be more effective at blocking acute hippocampal neural injury and subsequent neuroinflammation than it is to stop SRS activity in epilepsy, especially in refractory epilepsy. This is because TLE, and epilepsy in general, has a complex pathophysiology while acute hippocampal neural injury is largely due to excitotoxic synaptic Glu release that results in microglial activation and astrogliosis.

Riluzole is not currently used in patients following acute brain injury in the clinic as it may not be clear what may be a first SE event *versus* chronic epilepsy. We propose that riluzole could be used as a preventative strategy to limit excitotoxic neural injury in the hippocampus and extralimbic regions following an initial brain injury or SE event to suppress the neuroimmune response that is believed to initiate epileptogenesis. Riluzole here at 10 mg/kg could not completely prevent spontaneous generalized tonic-clonic seizures observed after 3 months in all rats but significantly reduces the number of these SE events (>60%) compared to KA + vehicle-treated rats, which do develop generalized SRS in all rats. A higher acute dose of riluzole would potentially have greater anticonvulsant and neuroprotective properties but also would have more side effects (Coleman et al., 2015); although its short term use could prove to be more effective to prevent epileptogenesis in *all* rats in the KA-model of TLE. SE is an emergency situation in humans and first responders are generally present soon after an SE event is reported. While administration of riluzole 1 h post SE attenuates hippocampal neural injury in the KA model (Kyllo et al., 2023), it is not yet known how late one can administer riluzole after SE to reduce acute hippocampal neural injury and the neuroimmune response in this model. Thirty minutes of SE is required in the pilocarpine model to produce acute hippocampal neural injury (Colvolan and Mello, 2000; Brandt et al., 2015). Another limitation of this study is the lack of electroencephalogram (EEG) recordings to monitor seizure activity. Future studies will require the use of EEG recordings for continuous monitoring in the brain so that focal seizure events can also be assessed in addition to observable recurrent generalized seizure activity reported here.

In the present study we showed that blocking acute hippocampal neural injury by riluzole attenuates the subsequent neuroinflammatory response up to 2 weeks post-SE and therefore, riluzole has significant potential as an antiepileptogenic agent to prevent epileptogenesis in the KA model of TLE. We also report that riluzole treatment after KA-induced SE attenuated hippocampal-dependent cognitive deficits, behavioral hyperexcitability, and spontaneous generalized SE activity after 3 months. Therefore, riluzole has significant potential as an antiepileptogenic drug in the clinical setting following an initial SE event. Whether riluzole has potential antiepileptogenic property in other animal models of chemical-induced epilepsy including pilocarpine- or diisopropylfluorophosphate-induced SE or following traumatic brain injury that leads to post-traumatic epilepsy is not known. Similar to riluzole, a number of novel benzothiazole compounds are reported potent anticonvulsants (Jimonet et al., 1999; Ugale et al., 2012; Ali and Siddiqui, 2013; Jagbir et al., 2015; Coleman et al., 2015) but have not been evaluated as neuroprotective *nor* as potential antiepileptogenic drugs. Together, the results presented in this study indicate that riluzole is a potential antiepileptogenic agent in the KA model of TLE and suggest that riluzole should be considered as preventative therapy after an acute brain injury such as SE to prevent the development of epilepsy.

## Data Availability

The raw data supporting the conclusions of this article will be made available by the authors, without undue reservation. Further summary data and statistical analyses are found in the Supplementary Material.

## Supplementary material

SUPPLEMENTARY FIGURE S1 Riluzole attenuates neural degeneration in the mediodorsal thalamus caused by KA-induced SE. Riluzole-treated rats showed less FJC labeling (green) than the KA group in the mediodorsal thalamus at 7 days (top row) and 14 days (bottom row). *Indicates the 3rd ventricle. Low power (200x) and high power (400x) was captured using an exposure time of 400 ms. Scale bar = 200 μM for low power and 50 μM for high power.

## Abbreviations

AMPAR, AMPA Glu receptor; ALS, Amyotrophic lateral sclerosis; ANOVA, Analysis of variance; CTCF, Corrected total cell fluorescence; DAPI, 4′,6-diamidino-2-phenylindole; DG, Dentate gyrus; EEG, Electroencephalogram; EAAT2, Excitatory amino acid transporter 2; FJC, Fluoro-jade C; Glu, Glutamate; Glt1, Glutamate transporter-1; Gln, Glutamine; i.p., Intraperitoneal; DG/CA4, Dendate hilar region; KA, Kainic acid; NARGT, Neural activity-regulated glutamine transporter; NMDAR, N-methyl-D-aspartate Glu receptor; NOR, Novel object recognition; PFA, Paraformaldehyde; I_NaP_, Persistent sodium current; PBS, Phosphate buffered saline; ROI, Regions of interest; Ril, Riluzole; SRS, Spontaneous recurrent seizures; SE, Status epilepticus; TLE, Temporal lobe epilepsy; Ca_V_, Voltage-gated calcium channel; Na_V_, Voltage-gated sodium channel.
