# Corrigendum: Riluzole attenuates acute neural injury and reactive gliosis, hippocampal-dependent cognitive impairments and spontaneous recurrent generalized seizures in a rat model of temporal lobe epilepsy

**DOI:** 10.3389/fphar.2025.1592653

**Published:** 2025-03-31

**Authors:** Thomas Kyllo, Dominic Allocco, Laine Vande Hei, Heike Wulff, Jeffrey D. Erickson

**Affiliations:** ^1^ Neuroscience Center of Excellence, School of Medicine, Louisiana State University Health-New Orleans, New Orleans, LA, United States; ^2^ Department of Pharmacology, School of Medicine, University of California-Davis, Davis, CA, United States

**Keywords:** acute neural injury, kainic acid, neuroprotection, neuroinflammatory response, microglia and astrocytes, epileptogenesis, antiepileptogenic drug

In the published article, the legend of **Supplementary Figure S1** was mistakenly not included in the publication. The missing legend appears below:

“**SUPPLEMENTARY FIGURE S1** Riluzole attenuates neural degeneration in the mediodorsal thalamus caused by KA-induced SE. Riluzole-treated rats showed less FJC labeling (*green*) than the KA group in the mediodorsal thalamus at 7 days (*top row*) and 14 days (*bottom row*). *Indicates the 3^rd^ ventricle. Low power (200x) and high power (400x) was captured using an exposure time of 400 ms. Scale bar = 200 μM for low power and 50 μM for high power.”

The authors apologize for this error and state that this does not change the scientific conclusions of the article in any way. The original article has been updated.

