# Supplementary figures and images for: Riluzole attenuates acute neural injury and reactive gliosis, hippocampal-dependent cognitive impairments and spontaneous recurrent generalized seizures in a rat model of temporal lobe epilepsy
Source: Front Pharmacol. 2024 Oct 30;15:1466953. doi: 10.3389/fphar.2024.1466953 (PMC11558044; doi:10.3389/fphar.2024.1466953)

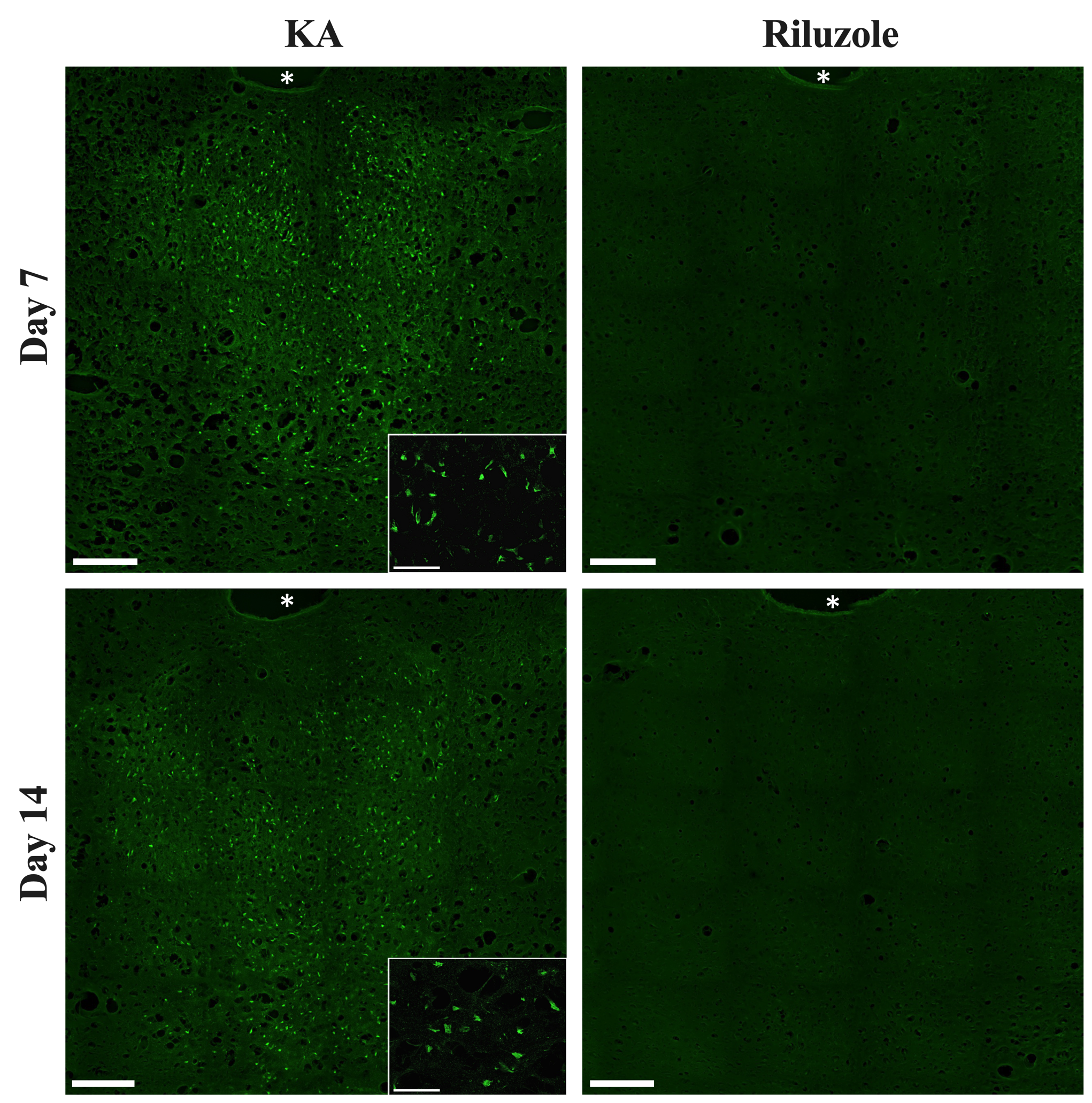

Supplement: Supplementary file 1 [file Image1.TIFF]
