# Supplementary material for: Riluzole attenuates acute neural injury and reactive gliosis, hippocampal-dependent cognitive impairments and spontaneous recurrent generalized seizures in a rat model of temporal lobe epilepsy
Source: Front Pharmacol. 2024 Oct 30;15:1466953. doi: 10.3389/fphar.2024.1466953 (PMC11558044; doi:10.3389/fphar.2024.1466953)
Supplement: Supplementary file 2 [file Table1.DOCX]

| **Table 1:** Fluoro jade C (Day 3) | | | | | | | | | |
| --- | --- | --- | --- | --- | --- | --- | --- | --- | --- |
|  |  | |  | |  | | | CI | |
|  | n | mean | | SD | | SEM | Lower | | Upper |
| Hippocampus |  |  | |  | |  |  | |  |
| Sham | 4 | 18534 | | 13400 | | 6700 | -2789 | | 39857 |
| KA + vehicle | 6 | 363500 | | 276584 | | 112915 | 73243 | | 653757 |

| **Table 2:** Fluoro jade C (Day 7) | | | | | | | | | |  |  | |
| --- | --- | --- | --- | --- | --- | --- | --- | --- | --- | --- | --- | --- |
|  |  |  | |  | | |  | | CI | | | |
|  | n | | mean | | SD | SEM | | Lower | | | | Upper |
| Hippocampus |  | |  | |  |  | |  | | | |  |
| Sham | 6 | | 29047 | | 13021 | 5316 | | 15383 | | | | 42712 |
| KA + vehicle | 7 | | 387498 | | 145846 | 55124 | | 252613 | | | | 522382 |
| KA + riluzole | 5 | | 42180 | | 26145 | 11692 | | 9718 | | | | 74643 |
| CA3 |  | |  | |  |  | |  | | | |  |
| Sham | 6 | | 312.0 | | 301.9 | 123.2 | | -4.832 | | | | 628.8 |
| KA + vehicle | 7 | | 62793 | | 18590 | 7026 | | 450600 | | | | 79986 |
| KA + riluzole | 5 | | 9275 | | 12260 | 5483 | | -5948 | | | | 24497 |
| CA1 |  | |  | |  |  | |  | | | |  |
| Sham | 6 | | 1839 | | 2115 | 863.4 | | -380.8 | | | | 4058 |
| KA + vehicle | 7 | | 53231 | | 23212 | 8773 | | 31763 | | | | 74699 |
| KA + riluzole | 5 | | 1133 | | 1115 | 498.8 | | -251.8 | | | | 2518 |
| CA4/H |  | |  | |  |  | |  | | | |  |
| Sham | 6 | | 1175 | | 2168 | 885.2 | | -1100 | | | | 3450 |
| KA + vehicle | 7 | | 74279 | | 31025 | 11726 | | 45586 | | | | 102972 |
| KA + riluzole | 5 | | 287.1 | | 374.5 | 167.5 | | -177.9 | | | | 752.1 |

| **Table 3:** Fluoro jade C (Day 14) | | | | | | | | |  |  | |
| --- | --- | --- | --- | --- | --- | --- | --- | --- | --- | --- | --- |
|  |  |  | |  | | |  | | CI | | |
|  | n | | mean | | SD | SEM | | Lower | | | Upper |
| Hippocampus |  | |  | |  |  | |  | | |  |
| Sham | 6 | | 26289 | | 13749 | 5613 | | 11861 | | | 40717 |
| KA + vehicle | 8 | | 301851 | | 148010 | 52329 | | 178111 | | | 425590 |
| KA + riluzole | 5 | | 25693 | | 17365 | 7766 | | 4131 | | | 47255 |
| CA3 |  | |  | |  |  | |  | | |  |
| Sham | 6 | | 275.2 | | 390.3 | 159.4 | | -134.4 | | | 684.8 |
| KA + vehicle | 8 | | 39714 | | 32232 | 11396 | | 12767 | | | 66660 |
| KA + riluzole | 5 | | 208.8 | | 122.5 | 54.79 | | 56.67 | | | 360.9 |
| CA1 |  | |  | |  |  | |  | | |  |
| Sham | 6 | | 216.5 | | 384.8 | 157.1 | | -187.3 | | | 620.3 |
| KA + vehicle | 8 | | 49464 | | 35640 | 12601 | | 19669 | | | 79260 |
| KA + riluzole | 5 | | 1405 | | 2118 | 947.0 | | -1224 | | | 4034 |
| CA4/H |  | |  | |  |  | |  | | |  |
| Sham | 6 | | 117.0 | | 108.7 | 44.40 | | 2.904 | | | 231.2 |
| KA + vehicle | 8 | | 47628 | | 50381 | 17812 | | 5509 | | | 89748 |
| KA + riluzole | 5 | | 2196 | | 2690 | 1203 | | -1144 | | | 5536 |

| **Table 4:** NeuN (Day 3) | | | | | |  | |  |
| --- | --- | --- | --- | --- | --- | --- | --- | --- |
|  |  |  |  |  | CI | | | |
|  | n | mean | SD | SEM | Lower | | Upper | |
| Hippocampus |  |  |  |  |  | |  | |
| Sham | 4 | 3074139 | 310783 | 155391 | 2579614 | | 3568663 | |
| KA + vehicle | 6 | 1214638 | 368199 | 150317 | 828237 | | 1601039 | |

| **Table 5:** NeuN (Day 7) | | | | | |  |  | |
| --- | --- | --- | --- | --- | --- | --- | --- | --- |
|  |  |  |  |  | CI | | | |
|  | n | mean | SD | SEM | Lower | | | Upper |
| Hippocampus |  |  |  |  |  | | |  |
| Sham | 6 | 3108565 | 401808 | 164038 | 2686893 | | | 3530238 |
| KA + vehicle | 7 | 913411 | 562121 | 212462 | 393536 | | | 1433286 |
| KA + riluzole | 5 | 3198648 | 497755 | 222603 | 2580604 | | | 3816692 |
| CA3 |  |  |  |  |  | | |  |
| Sham | 6 | 1003082 | 178974 | 73066 | 815260 | | | 1190904 |
| KA + vehicle | 7 | 344682 | 137481 | 51963 | 217534 | | | 471831 |
| KA + riluzole | 5 | 1000443 | 218545 | 97736 | 729084 | | | 1271802 |
| CA1 |  |  |  |  |  | | |  |
| Sham | 6 | 824124 | 184954 | 75507 | 630026 | | | 1018221 |
| KA + vehicle | 7 | 340772 | 322680 | 121962 | 42343 | | | 639201 |
| KA + riluzole | 5 | 919254 | 101874 | 45559 | 792761 | | | 1045747 |
| CA4/H |  |  |  |  |  | | |  |
| Sham | 6 | 1281360 | 141387 | 57721 | 1132983 | | | 1429736 |
| KA + vehicle | 7 | 227956 | 165483 | 62547 | 74910 | | | 381003 |
| KA + riluzole | 5 | 1278950 | 271502 | 121419 | 941837 | | | 1616064 |

| **Table 6:** NeuN (Day 14) | | | | | |  |  | |
| --- | --- | --- | --- | --- | --- | --- | --- | --- |
|  |  |  |  |  | CI | | | |
|  | n | mean | SD | SEM | Lower | | | Upper |
| Hippocampus |  |  |  |  |  | | |  |
| Sham | 6 | 2958320 | 423249 | 172790 | 2514148 | | | 3402492 |
| KA + vehicle | 8 | 1119456 | 615448 | 217594 | 604929 | | | 1633983 |
| KA + riluzole | 5 | 3230107 | 118942 | 53193 | 3082421 | | | 3377793 |
| CA3 |  |  |  |  |  | | |  |
| Sham | 6 | 1110937 | 231284 | 94421 | 868219 | | | 1353654 |
| KA + vehicle | 8 | 435112 | 218913 | 77398 | 252096 | | | 618128 |
| KA + riluzole | 5 | 1004412 | 190066 | 85000 | 768413 | | | 1240410 |
| CA1 |  |  |  |  |  | | |  |
| Sham | 6 | 744829 | 139080 | 56779 | 598873 | | | 890784 |
| KA + vehicle | 8 | 247025 | 238001 | 84146 | 48052 | | | 445999 |
| KA + riluzole | 5 | 884133 | 75065 | 33570 | 790927 | | | 977339 |
| CA4/H |  |  |  |  |  | | |  |
| Sham | 6 | 1102554 | 420601 | 171710 | 661160 | | | 1543948 |
| KA + vehicle | 8 | 437319 | 319125 | 112828 | 170523 | | | 704114 |
| KA + riluzole | 5 | 1341563 | 124774 | 55801 | 1186635 | | | 1496490 |

| **Table 7:** Iba1 (Day 3) | | | | | |  |  | |
| --- | --- | --- | --- | --- | --- | --- | --- | --- |
|  |  |  |  |  | CI | | | |
|  | n | mean | SD | SEM | Lower | | | Upper |
| Hippocampus |  |  |  |  |  | | |  |
| Sham | 4 | 634891 | 353867 | 176933 | 71810 | | | 1197972 |
| KA + vehicle | 6 | 7192076 | 1894284 | 773338 | 5204146 | | | 9180005 |

| **Table 8:** Iba1 (Day 7) | | | | | |  | |  |
| --- | --- | --- | --- | --- | --- | --- | --- | --- |
|  |  |  |  |  | CI | | | |
|  | n | mean | SD | SEM | Lower | | Upper | |
| Hippocampus |  |  |  |  |  | |  | |
| Sham | 6 | 512507 | 640279 | 261393 | -159424 | | 1184439 | |
| KA + vehicle | 7 | 20300329 | 4352345 | 1645032 | 16275082 | | 24325577 | |
| KA + riluzole | 5 | 2464272 | 2381551 | 1065062 | -492814 | | 5421358 | |
| CA3 |  |  |  |  |  | |  | |
| Sham | 6 | 52001 | 25029 | 10218 | 25735 | | 78268 | |
| KA + vehicle | 7 | 2413937 | 607800 | 229727 | 1851816 | | 2976059 | |
| KA + riluzole | 5 | 818685 | 671185 | 300163 | -14701 | | 1652072 | |
| CA1 |  |  |  |  |  | |  | |
| Sham | 6 | 68185 | 62054 | 25333 | 3064 | | 133307 | |
| KA + vehicle | 7 | 2076902 | 471010 | 178025 | 1641290 | | 2512513 | |
| KA + riluzole | 5 | 178412 | 174152 | 77883 | -37827 | | 394651 | |
| CA4/H |  |  |  |  |  | |  | |
| Sham | 6 | 128155 | 77367 | 31585 | 46963 | | 209347 | |
| KA + vehicle | 7 | 2550877 | 482109 | 182220 | 2105001 | | 2996753 | |
| KA + riluzole | 5 | 263452 | 128083 | 57281 | 104416 | | 422488 | |

| **Table 9:** Iba1 (Day 14) | | | | | |  | |  |
| --- | --- | --- | --- | --- | --- | --- | --- | --- |
|  |  |  |  |  | CI | | | |
|  | n | mean | SD | SEM | Lower | | Upper | |
| Hippocampus |  |  |  |  |  | |  | |
| Sham | 6 | 976903 | 541536 | 221081 | 408595 | | 1545210 | |
| KA + vehicle | 8 | 15564920 | 7243891 | 2561102 | 9508876 | | 21620965 | |
| KA + riluzole | 5 | 2149524 | 1594345 | 713013 | 169884 | | 4129164 | |
| CA3 |  |  |  |  |  | |  | |
| Sham | 6 | 80845 | 55493 | 22655 | 22608 | | 139082 | |
| KA + vehicle | 8 | 1555238 | 1097546 | 388041 | 637666 | | 2472809 | |
| KA + riluzole | 5 | 104318 | 31869 | 14252 | 64748 | | 143889 | |
| CA1 |  |  |  |  |  | |  | |
| Sham | 6 | 110746 | 89067 | 36362 | 17276 | | 204217 | |
| KA + vehicle | 8 | 2185241 | 1041737 | 368310 | 1314327 | | 3056154 | |
| KA + riluzole | 5 | 308722 | 269666 | 120598 | -26113 | | 643557 | |
| CA4/H |  |  |  |  |  | |  | |
| Sham | 6 | 188419 | 126639 | 51700 | 55519 | | 321318 | |
| KA + vehicle | 8 | 1515415 | 1162248 | 410917 | 543751 | | 2487079 | |
| KA + riluzole | 5 | 299790 | 214930 | 96120 | 32919 | | 566661 | |

| **Table 10:** ED-1 (Day 3) | | | | | |  |  | |
| --- | --- | --- | --- | --- | --- | --- | --- | --- |
|  |  |  |  |  | CI | | | |
|  | n | mean | SD | SEM | Lower | | | Upper |
| Hippocampus |  |  |  |  |  | | |  |
| Sham | 4 | 29142 | 12307 | 6154 | 9558 | | | 48725 |
| KA + vehicle | 6 | 244718 | 100018 | 50009 | 85567 | | | 403868 |

| **Table 11:** ED-1 (Day 7) | | | | | |  | |  |
| --- | --- | --- | --- | --- | --- | --- | --- | --- |
|  |  |  |  |  | CI | | | |
|  | n | mean | SD | SEM | Lower | | Upper | |
| Hippocampus |  |  |  |  |  | |  | |
| Sham | 6 | 75868 | 36806 | 15026 | 37243 | | 114493 | |
| KA + vehicle | 7 | 3650668 | 3362204 | 1270794 | 541147 | | 6760188 | |
| KA + riluzole | 5 | 94100 | 70760 | 31645 | 6240 | | 181959 | |
| CA3 |  |  |  |  |  | |  | |
| Sham | 6 | 1175 | 1205 | 491.8 | -89.00 | | 2439 | |
| KA + vehicle | 7 | 470191 | 377411 | 142648 | 121144 | | 819238 | |
| KA + riluzole | 5 | 3174 | 2725 | 1219 | -210.1 | | 6557 | |
| CA1 |  |  |  |  |  | |  | |
| Sham | 6 | 2506 | 1650 | 673.7 | 774.2 | | 4238 | |
| KA + vehicle | 7 | 306610 | 338306 | 12786 | -6271 | | 619491 | |
| KA + riluzole | 5 | 1542 | 1112 | 497.3 | 161.0 | | 2922 | |
| CA4/H |  |  |  |  |  | |  | |
| Sham | 6 | 2941 | 5378 | 2196 | -2704 | | 8585 | |
| KA + vehicle | 7 | 452225 | 463914 | 175343 | 23176 | | 881274 | |
| KA + riluzole | 5 | 2398 | 2814 | 1259 | -1096 | | 5892 | |

| **Table 12:** ED-1 (Day 14) | | | | | | |
| --- | --- | --- | --- | --- | --- | --- |
|  |  |  |  |  | CI | |
|  | n | mean | SD | SEM | Lower | Upper |
| Hippocampus |  |  |  |  |  |  |
| Sham | 6 | 40333 | 26326 | 10747 | 12706 | 67961 |
| KA + vehicle | 8 | 4319161 | 4170593 | 1474527 | 832458 | 7805864 |
| KA + riluzole | 5 | 52184 | 8099 | 3622 | 42127 | 62241 |
| CA3 |  |  |  |  |  |  |
| Sham | 6 | 1573 | 2561 | 1046 | -1114 | 4261 |
| KA + vehicle | 8 | 336847 | 294726 | 104201 | 90450 | 583244 |
| KA + riluzole | 5 | 3723 | 4151 | 1856 | -1431 | 8877 |
| CA1 |  |  |  |  |  |  |
| Sham | 6 | 1460 | 1521 | 621.1 | -136.8 | 3056 |
| KA + vehicle | 8 | 682421 | 742055 | 262356 | 62048 | 1302795 |
| KA + riluzole | 5 | 2220 | 2686 | 1201 | -1114 | 5555 |
| CA4/H |  |  |  |  |  |  |
| Sham | 6 | 1815 | 2346 | 957.8 | -647.3 | 4277 |
| KA + vehicle | 8 | 405761 | 420663 | 148727 | 54077 | 757444 |
| KA + riluzole | 5 | 1032 | 1046 | 467.8 | -266.4 | 2331 |

| **Table 13:** GFAP (Day 3) | | | | | |  | |  |
| --- | --- | --- | --- | --- | --- | --- | --- | --- |
|  |  |  |  |  | CI | | | |
|  | n | mean | SD | SEM | Lower | | Upper | |
| Hippocampus |  |  |  |  |  | |  | |
| Sham | 4 | 3686177 | 2016635 | 1008318 | 477260 | | 6895094 | |
| KA + vehicle | 6 | 6447672 | 3937208 | 1760773 | 1558983 | | 11336362 | |

| **Table 14:** GFAP (Day 7) | | | | | | |
| --- | --- | --- | --- | --- | --- | --- |
|  |  |  |  |  | CI | |
|  | n | mean | SD | SEM | Lower | Upper |
| Hippocampus |  |  |  |  |  |  |
| Sham | 6 | 3955990 | 2605612 | 1063737 | 1221567 | 6690412 |
| KA + vehicle | 7 | 19350454 | 5360775 | 2026182 | 14392564 | 24308344 |
| KA + riluzole | 5 | 6986114 | 2332158 | 1042973 | 4090357 | 9881870 |
| CA3 |  |  |  |  |  |  |
| Sham | 6 | 226658 | 199741 | 81544 | 17042 | 436273 |
| KA + vehicle | 7 | 1600111 | 683120 | 258195 | 968331 | 2231892 |
| KA + riluzole | 5 | 359480 | 271955 | 121622 | 21804 | 697156 |
| CA1 |  |  |  |  |  |  |
| Sham | 6 | 208771 | 154959 | 63262 | 46151 | 371391 |
| KA + vehicle | 7 | 1716764 | 393578 | 148758 | 1352766 | 2080763 |
| KA + riluzole | 5 | 699445 | 125201 | 55991 | 543987 | 854902 |
| CA4/H |  |  |  |  |  |  |
| Sham | 6 | 993912 | 125367 | 51181 | 862348 | 1125477 |
| KA + vehicle | 7 | 1647874 | 560109 | 211701 | 1129860 | 2165889 |
| KA + riluzole | 5 | 1181987 | 506345 | 226444 | 553276 | 1810697 |

| **Table 15:** GFAP (Day 14) | | | | | | |
| --- | --- | --- | --- | --- | --- | --- |
|  |  |  |  |  | CI | |
|  | n | mean | SD | SEM | Lower | Upper |
| Hippocampus |  |  |  |  |  |  |
| Sham | 6 | 2622525 | 1428496 | 583181 | 1123411 | 4121640 |
| KA + vehicle | 8 | 25879689 | 3894835 | 1377032 | 22623525 | 29135853 |
| KA + riluzole | 5 | 5956510 | 1941040 | 868059 | 3546391 | 8366629 |
| CA3 |  |  |  |  |  |  |
| Sham | 6 | 163180 | 166345 | 67910 | -11389 | 337749 |
| KA + vehicle | 8 | 1569868 | 392028 | 138603 | 1242124 | 1897612 |
| KA + riluzole | 5 | 446924 | 101269 | 45289 | 321183 | 45289 |
| CA1 |  |  |  |  |  |  |
| Sham | 6 | 407764 | 248988 | 101649 | 146467 | 669061 |
| KA + vehicle | 8 | 2487012 | 587137 | 207584 | 1996153 | 2977871 |
| KA + riluzole | 5 | 501504 | 302397 | 135236 | 126029 | 876979 |
| CA4/H |  |  |  |  |  |  |
| Sham | 6 | 759200 | 261038 | 106568 | 485257 | 1033143 |
| KA + vehicle | 8 | 2650909 | 919636 | 325141 | 1882074 | 3419744 |
| KA + riluzole | 5 | 1198217 | 553658 | 247603 | 510759 | 1885674 |

| **Table 16:** Vimentin (Day 3) | | | | |  |  |
| --- | --- | --- | --- | --- | --- | --- |
|  |  |  |  |  | CI | |
|  | n | mean | SD | SEM | Lower | Upper |
| Hippocampus |  |  |  |  |  |  |
| Sham | 4 | 222212 | 73343 | 36672 | 105506 | 338917 |
| KA + vehicle | 6 | 1813024 | 283680 | 115812 | 1515321 | 2110728 |

| **Table 17:** Vimentin (Day 7) | | | | | | | |
| --- | --- | --- | --- | --- | --- | --- | --- |
|  |  |  |  |  | CI | |  |
|  | n | mean | SD | SEM | Lower | Upper |  |
| Hippocampus |  |  |  |  |  |  |  |
| Sham | 6 | 158830 | 114127 | 46592 | 39060 | 278599 |  |
| KA + vehicle | 7 | 3970165 | 2681176 | 1013389 | 1490491 | 6449839 |  |
| KA + riluzole | 5 | 627504 | 763189 | 341309 | -320120 | 1575129 |  |
| CA3 |  |  |  |  |  |  |  |
| Sham | 6 | 3057 | 2903 | 1185 | 10.81 | 6103 |  |
| KA + vehicle | 7 | 208493 | 146979 | 55553 | 72560 | 344426 |  |
| KA + riluzole | 5 | 19835 | 25464 | 11388 | -11782 | 51453 |  |
| CA1 |  |  |  |  |  |  |  |
| Sham | 6 | 2737 | 1857 | 758.3 | 788.2 | 4687 |  |
| KA + vehicle | 7 | 480455 | 418894 | 158327 | 93042 | 867867 |  |
| KA + riluzole | 5 | 74606 | 149462 | 66842 | -110976 | 260188 |  |
| CA4/H |  |  |  |  |  |  |  |
| Sham | 6 | 2918 | 3902 | 1593 | -1177 | 7013 |  |
| KA + vehicle | 7 | 225782 | 138871 | 52488 | 97348 | 354216 |  |
| KA + riluzole | 5 | 11039 | 13676 | 6116 | -5942 | 28020 |  |

| **Table 18:** Vimentin (Day 14) | | | | | | | |
| --- | --- | --- | --- | --- | --- | --- | --- |
|  |  |  |  |  | CI | |  |
|  | n | mean | SD | SEM | Lower | Upper |  |
| Hippocampus |  |  |  |  |  |  |  |
| Sham | 6 | 239585 | 55142 | 22512 | 181717 | 297453 |  |
| KA + vehicle | 8 | 12302313 | 5783570 | 2044801 | 7467127 | 17137498 |  |
| KA + riluzole | 5 | 879929 | 139629 | 62444 | 706557 | 1053301 |  |
| CA3 |  |  |  |  |  |  |  |
| Sham | 6 | 13015 | 20023 | 8175 | -7999 | 34028 |  |
| KA + vehicle | 8 | 631425 | 298862 | 105664 | 381570 | 881280 |  |
| KA + riluzole | 5 | 11964 | 16166 | 7230 | -8109 | 32036 |  |
| CA1 |  |  |  |  |  |  |  |
| Sham | 6 | 20921 | 21744 | 8877 | -1897 | 43740 |  |
| KA + vehicle | 8 | 1455144 | 873878 | 308963 | 724564 | 2185725 |  |
| KA + riluzole | 5 | 18490 | 17330 | 7750 | -3027 | 40008 |  |
| CA4/H |  |  |  |  |  |  |  |
| Sham | 6 | 8291 | 9294 | 3794 | -1463 | 18045 |  |
| KA + vehicle | 8 | 802410 | 668527 | 236360 | 243508 | 1361312 |  |
| KA + riluzole | 5 | 16669 | 8226 | 3679 | 6455 | 26883 |  |

| **Table 19:** Y maze | | | | | | | | | |  |
| --- | --- | --- | --- | --- | --- | --- | --- | --- | --- | --- |
|  |  |  |  | |  | | CI | | | |
|  | n | mean | | SD | | SEM | | Lower | Upper | |
| Spont. Alternation % |  |  | |  | |  | |  |  | |
| Sham | 14 | 69.85 | | 7.737 | | 2.068 | | 65.39 | 74.32 | |
| KA + vehicle | 16 | 46.36 | | 23.93 | | 5.981 | | 33.61 | 59.11 | |
| KA + riluzole | 13 | 61.63 | | 9.821 | | 2.724 | | 55.69 | 67.56 | |
| # of Arm Entries |  |  | |  | |  | |  |  | |
| Sham | 14 | 11.83 | | 2.050 | | 0.5478 | | 10.65 | 13.02 | |
| KA + vehicle | 16 | 11.17 | | 6.952 | | 1.738 | | 7.462 | 14.87 | |
| KA + riluzole | 13 | 12.90 | | 2.767 | | 0.7674 | | 11.23 | 14.57 | |
| # of Alternations |  |  | |  | |  | |  |  | |
| Sham | 14 | 6.905 | | 1.722 | | 0.4602 | | 5.911 | 7.899 | |
| KA + vehicle | 16 | 5.281 | | 4.911 | | 1.228 | | 2.664 | 7.898 | |
| KA + riluzole | 13 | 6.846 | | 2.053 | | 0.5694 | | 5.605 | 8.087 | |

| **Table 20:** Behavioral Hyperexcitability Test | | | | |  | |  | |
| --- | --- | --- | --- | --- | --- | --- | --- | --- |
|  |  |  |  |  | | CI | | |
|  | n | mean | SD | SEM | | Lower | | Upper |
| Approach-response test |  |  |  |  | |  | |  |
| Sham | 14 | 1.786 | 0.4258 | 0.1138 | | 1.540 | | 2.032 |
| KA + vehicle | 16 | 2.250 | 1.125 | 0.2814 | | 1.650 | | 2.850 |
| KA + riluzole | 13 | 1.692 | 0.4804 | 0.1332 | | 1.402 | | 1.983 |
| Touch-response  test |  |  |  |  | |  | |  |
| Sham | 14 | 1.357 | 0.7449 | 0.1991 | | 0.9270 | | 1.787 |
| KA + vehicle | 16 | 3.250 | 2.236 | 0.5590 | | 2.058 | | 4.442 |
| KA + riluzole | 13 | 1.538 | 1.450 | 0.4022 | | 0.6622 | | 2.415 |
| Finger-snap  test |  |  |  |  | |  | |  |
| Sham | 14 | 1.286 | 0.4688 | 0.1253 | | 1.015 | | 1.556 |
| KA + vehicle | 16 | 2.375 | 0.5000 | 0.1250 | | 2.109 | | 2.641 |
| KA + riluzole | 13 | 1.846 | 0.3755 | 0.1042 | | 1.619 | | 2.073 |
| Pick-up  test |  |  |  |  | |  | |  |
| Sham | 14 | 1.500 | 0.5189 | 0.1387 | | 1.200 | | 1.800 |
| KA + vehicle | 16 | 4.500 | 1.461 | 0.3651 | | 3.722 | | 5.278 |
| KA + riluzole | 13 | 2.231 | 1.691 | 0.4690 | | 1.209 | | 3.253 |
| Behavioral Hyperexcitability Score |  |  |  |  | |  | |  |
| Sham | 14 | 5.929 | 1.542 | 0.4122 | | 5.038 | | 6.819 |
| KA + vehicle | 16 | 12.38 | 3.775 | 0.9437 | | 10.36 | | 14.39 |
| KA + riluzole | 13 | 7.308 | 1.974 | 0.5475 | | 6.115 | | 8.501 |

| **Table 21:** Spontaneous Generalized Recurrent Seizure  Activity | | | | |  | |  | |
| --- | --- | --- | --- | --- | --- | --- | --- | --- |
|  |  |  |  |  | | CI | | |
|  | n | mean | SD | SEM | | Lower | | Upper |
| Sham | 14 | 0.000 | 0.000 | 0.000 | | 0.000 | | 0.000 |
| KA + vehicle | 16 | 10.78 | 8.363 | 2.091 | | 6.325 | | 15.24 |
| KA + riluzole | 12 | 3.917 | 3.895 | 1.125 | | 1.442 | | 6.392 |

| **Table 22:** % Weight Change | | | | |  |  |
| --- | --- | --- | --- | --- | --- | --- |
|  |  |  |  |  | CI | |
|  | n | mean | SD | SEM | Lower | Upper |
| Day 0 |  |  |  |  |  |  |
| Sham | 24 | 0.000 | 0.000 | 0.000 | 0.000 | 0.000 |
| KA + vehicle | 28 | 0.000 | 0.000 | 0.000 | 0.000 | 0.000 |
| KA + riluzole | 21 | 0.000 | 0.000 | 0.000 | 0.000 | 0.000 |
| Day 1 |  |  |  |  |  |  |
| Sham | 24 | 0.6065 | 1.017 | 0.208 | 0.181 | 1.039 |
| KA + vehicle | 28 | -14.55 | 3.650 | 0.690 | -15.968 | -13.137 |
| KA + riluzole | 21 | -11.52 | 3.943 | 0.860 | -13.324 | -9.735 |
| Day 2 |  |  |  |  |  |  |
| Sham | 24 | 2.723 | 1.754 | 0.358 | 1.994 | 3.476 |
| KA + vehicle | 28 | -19.29 | 5.296 | 1.001 | -21.341 | -17.234 |
| KA + riluzole | 21 | -10.93 | 6.755 | 1.474 | -14.015 | -7.865 |
| Day 3 |  |  |  |  |  |  |
| Sham | 24 | 4.377 | 1.854 | 0.378 | 3.622 | 5.188 |
| KA + vehicle | 28 | -20.68 | 7.020 | 1.327 | -23.402 | -17.957 |
| KA + riluzole | 21 | -9.049 | 6.257 | 1.365 | -11.892 | -6.196 |
| Day 4 |  |  |  |  |  |  |
| Sham | 24 | 6.185 | 1.737 | 0.355 | 5.483 | 6.950 |
| KA + vehicle | 28 | -22.75 | 8.353 | 1.579 | -25.991 | -19.513 |
| KA + riluzole | 21 | -6.184 | 5.879 | 1.283 | -8.863 | -3.511 |
| Day 5 |  |  |  |  |  |  |
| Sham | 24 | 7.582 | 1.828 | 0.373 | 6.801 | 8.345 |
| KA + vehicle | 28 | -22.50 | 10.466 | 1.978 | -26.556 | -18.439 |
| KA + riluzole | 21 | -3.738 | 5.256 | 1.147 | -6.197 | -1.411 |
| Day 6 |  |  |  |  |  |  |
| Sham | 24 | 9.420 | 2.515 | 0.513 | 8.325 | 10.449 |
| KA + vehicle | 28 | -21.30 | 12.338 | 2.332 | -26.081 | -16.513 |
| KA + riluzole | 21 | -1.192 | 4.393 | 0.959 | -3.191 | 0.808 |

| **Table 23:** NOR test | | |  | | | |
| --- | --- | --- | --- | --- | --- | --- |
|  |  |  |  |  | CI | |
| Acquisition  Phase | n | Mean | SD | SEM | Lower | Upper |
| Time (S) |  |  |  |  |  |  |
| Sham | 14 | Obj. 1: 22.071  Obj. 2: 20.143 | Obj. 1: 17.063  Obj. 2: 11.707 | Obj. 1: 4.560  Obj. 2: 3.129 | Obj. 1: 12.220  Obj. 2: 13.383 | Obj. 1: 31.923  Obj. 2: 26.902 |
| KA + vehicle | 16 | Obj. 1: 14.750  Obj. 2: 11.313 | Obj. 1: 11.693  Obj. 2: 11.453 | Obj. 1: 2.923  Obj. 2: 2.863 | Obj. 1: 8.519  Obj. 2: 5.210 | Obj. 1: 20.981  Obj. 2: 17.415 |
| KA + riluzole | 12 | Obj. 1: 14.917  Obj. 2: 13.917 | Obj. 1: 16.692  Obj. 2: 14.551 | Obj. 1: 4.819  Obj. 2: 4.200 | Obj. 1: 4.311  Obj. 2: 4.672 | Obj. 1: 25.522  Obj. 2: 23.162 |
| % of Time |  |  |  |  |  |  |
| Sham | 14 | Obj. 1: 50.792  Obj. 2: 49.208 | Obj. 1: 8.128  Obj. 2: 8.128 | Obj. 1: 2.172  Obj. 2: 2.172 | Obj. 1: 46.099  Obj. 2: 44.515 | Obj. 1: 55.485  Obj. 2: 53.901 |
| KA + vehicle | 14 | Obj. 1: 58.036  Obj. 2: 41.964 | Obj. 1: 24.130  Obj. 2: 24.130 | Obj. 1: 6.449  Obj. 2: 6.449 | Obj. 1: 44.103  Obj. 2: 28.032 | Obj. 1: 71.968  Obj. 2: 55.897 |
| KA + riluzole | 11 | Obj. 1: 49.049  Obj. 2: 50.951 | Obj. 1: 16.727  Obj. 2: 16.727 | Obj. 1: 5.043  Obj. 2: 5.043 | Obj. 1: 37.812  Obj. 2: 39.713 | Obj. 1: 60.287  Obj. 2: 62.188 |
|  |  |  |  |  | CI | |
| Retrieval  Phase | n | Mean | SD | SEM | Lower | Upper |
| Time (S) |  |  |  |  |  |  |
| Sham | 14 | Obj. 2: 12.71  Novel Obj.: 19.79 | Obj. 2: 5.24  Novel Obj.: 10.06 | Obj. 2: 1.40  Novel Obj.: 2.69 | Obj. 2: 9.69  Novel Obj.: 13.98 | Obj. 2: 15.74  Novel Obj.: 25.59 |
| KA + vehicle | 16 | Obj. 2: 10.69  Novel Obj.: 12.06 | Obj. 2: 11.24  Novel Obj.: 10.94 | Obj. 2: 2.81  Novel Obj.: 2.74 | Obj. 2: 4.70  Novel Obj.: 6.23 | Obj. 2: 16.67  Novel Obj.: 17.89 |
| KA + riluzole | 12 | Obj. 2: 8.92  Novel Obj.: 14.50 | Obj. 2: 8.62  Novel Obj.: 14.87 | Obj. 2: 2.49  Novel Obj.: 4.29 | Obj. 2: 3.44  Novel Obj.: 5.05 | Obj. 2: 14.39  Novel Obj.: 23.95 |
| % of Time |  |  |  |  |  |  |
| Sham | 14 | Obj. 2: 41.74  Novel Obj.: 58.26 | Obj. 2: 13.59  Novel Obj.: 13.59 | Obj. 2: 3.63  Novel Obj.: 3.63 | Obj. 2: 33.90  Novel Obj.: 50.41 | Obj. 2: 49.59  Novel Obj.: 66.10 |
| KA + vehicle | 15 | Obj. 2: 42.37  Novel Obj.: 50.96 | Obj. 2: 17.18  Novel Obj.: 18.88 | Obj. 2: 4.44  Novel Obj.: 4.88 | Obj. 2: 32.86  Novel Obj.: 40.50 | Obj. 2: 51.89  Novel Obj.: 61.42 |
| KA + riluzole | 11 | Obj. 2: 40.79  Novel Obj.: 59.21 | Obj. 2: 9.50  Novel Obj.: 9.50 | Obj. 2: 2.86  Novel Obj.: 2.86 | Obj. 2: 34.41  Novel Obj.: 52.83 | Obj. 2: 47.17  Novel Obj.: 65.59 |

| **Table 24:** Barnes Maze Primary Latency | | | | | | | | | |  | | | |  |
| --- | --- | --- | --- | --- | --- | --- | --- | --- | --- | --- | --- | --- | --- | --- |
|  |  |  |  | | |  | | | CI | | | | | |
| Acquisition Phase | n | Mean (S) | | | SD | | | SEM | | | | Lower | Upper | |
| Day 1 |  |  | | |  | | |  | | | |  |  | |
| Sham | 14 | 69.893 | | | 52.008 | | | 13.900 | | | | 39.864 | 99.921 | |
| KA + vehicle | 16 | 118.594 | | | 54.932 | | | 13.733 | | | | 89.322 | 147.865 | |
| KA + riluzole | 13 | 80.115 | | | 54.732 | | | 15.180 | | | | 47.041 | 113.189 | |
| Day 2 |  |  | | |  | | |  | | | |  |  | |
| Sham | 14 | 34.379 | | | 28.253 | | | 7.551 | | | | 18.066 | 50.691 | |
| KA + vehicle | 16 | 116.000 | | | 67.715 | | | 16.929 | | | | 79.917 | 152.083 | |
| KA + riluzole | 13 | 46.346 | | | 50.268 | | | 13.942 | | | | 15.970 | 76.723 | |
| Day 3 |  |  | | |  | | |  | | | |  |  | |
| Sham | 14 | 27.464 | | | 25.744 | | | 6.880 | | | | 12.600 | 42.328 | |
| KA + vehicle | 16 | 114.750 | | | 67.008 | | | 16.752 | | | | 79.044 | 150.456 | |
| KA + riluzole | 13 | 33.692 | | | 46.132 | | | 12.795 | | | | 5.815 | 61.570 | |
| Day 4 |  |  | | |  | | |  | | | |  |  | |
| Sham | 14 | 21.393 | | | 15.044 | | | 4.021 | | | | 12.707 | 30.079 | |
| KA + vehicle | 16 | 108.656 | | | 68.326 | | | 17.081 | | | | 72.248 | 145.065 | |
| KA + riluzole | 13 | 44.192 | | | 61.379 | | | 17.024 | | | | 7.101 | 81.283 | |
|  |  |  | |  | | |  | | | | CI | | | |
| Probe Trials | n | Mean (S) | | | SD | | | SEM | | | | Lower | Upper | |
| Day 7 |  |  | | |  | | |  | | | |  |  | |
| Sham | 14 | 19.143 | | | 26.559 | | | 7.098 | | | | 3.808 | 34.477 | |
| KA + vehicle | 16 | 57.938 | | | 34.187 | | | 8.547 | | | | 39.721 | 76.154 | |
| KA + riluzole | 13 | 28.769 | | | 32.231 | | | 8.939 | | | | 9.292 | 48.246 | |
| Day 10 |  |  | | |  | | |  | | | |  |  | |
| Sham | 14 | 16.857 | | | 15.570 | | | 4.161 | | | | 7.867 | 25.847 | |
| KA + vehicle | 16 | 55.000 | | | 36.822 | | | 9.206 | | | | 35.379 | 74.621 | |
| KA + riluzole | 13 | 27.462 | | | 29.458 | | | 8.170 | | | | 9.660 | 45.263 | |

| **Table 25:** Barnes Maze Total Latency | | | | | | | | | |  | |  |
| --- | --- | --- | --- | --- | --- | --- | --- | --- | --- | --- | --- | --- |
|  |  | |  | |  | |  | | CI | | | |
| Acquisition Phase | n | Mean (S) | | SD | | SEM | | Lower | | | Upper | |
| Day 1 |  |  | |  | |  | |  | | |  | |
| Sham | 14 | 95.393 | | 46.235 | | 12.357 | | 68.697 | | | 122.088 | |
| KA + vehicle | 16 | 150.531 | | 41.514 | | 10.379 | | 128.410 | | | 172.653 | |
| KA + riluzole | 13 | 94.846 | | 59.308 | | 16.449 | | 59.007 | | | 130.686 | |
| Day 2 |  |  | |  | |  | |  | | |  | |
| Sham | 14 | 48.557 | | 45.431 | | 12.142 | | 22.326 | | | 74.788 | |
| KA + vehicle | 16 | 138.531 | | 59.213 | | 14.803 | | 106.979 | | | 170.083 | |
| KA + riluzole | 13 | 68.462 | | 65.576 | | 18.188 | | 28.834 | | | 108.089 | |
| Day 3 |  |  | |  | |  | |  | | |  | |
| Sham | 14 | 37.429 | | 34.378 | | 9.188 | | 17.579 | | | 57.278 | |
| KA + vehicle | 16 | 128.094 | | 60.602 | | 15.151 | | 95.801 | | | 160.386 | |
| KA + riluzole | 13 | 53.923 | | 62.447 | | 17.320 | | 91.659 | | | 16.187 | |
| Day 4 |  |  | |  | |  | |  | | |  | |
| Sham | 14 | 35.250 | | 31.519 | | 8.424 | | 17.051 | | | 53.449 | |
| KA + vehicle | 16 | 121.156 | | 70.523 | | 17.631 | | 83.577 | | | 158.735 | |
| KA + riluzole | 13 | 57.923 | | 62.432 | | 17.316 | | 20.196 | | | 95.651 | |
